# Characterization of Degenerate Configurations in Attitude Determination of Three-Vehicle Heterogeneous Formations

**DOI:** 10.3390/s21144631

**Published:** 2021-07-06

**Authors:** Pedro Cruz, Pedro Batista

**Affiliations:** 1 Laboratory for Robotics and Engineering Systems, Institute for Systems and Robotics, 1049-001 Lisboa, Portugal; pfcruz@isr.tecnico.ulisboa.pt; 2 Instituto Superior Técnico, Universidade de Lisboa, 1049-001 Lisboa, Portugal

**Keywords:** attitude determination, formation of vehicles, heterogeneous formation, ambiguous solution, degenerate solution

## Abstract

The existence of multiple solutions to an attitude determination problem impacts the design of estimation schemes, potentially increasing the errors by a significant value. It is therefore essential to identify such cases in any attitude problem. In this paper, the cases where multiple attitudes satisfy all constraints of a three-vehicle heterogeneous formation are identified. In the formation considered herein, the vehicles measure inertial references and relative line-of-sight vectors. Nonetheless, the line of sight between two elements of the formation is restricted, and these elements are denoted as deputies. The attitude determination problem is characterized relative to the number of solutions associated with each configuration of the formation. There are degenerate and ambiguous configurations that result in infinite or exactly two solutions, respectively. Otherwise, the problem has a unique solution. The degenerate configurations require some collinearity between independent measurements, whereas the ambiguous configurations result from symmetries in the formation measurements. The conditions which define all such configurations are determined in this work. Furthermore, the ambiguous subset of configurations is geometrically interpreted resorting to the planes defined by specific measurements. This subset is also shown to be a zero-measure subset of all possible configurations. Finally, a maneuver is simulated to illustrate and validate the conclusions. As a result of this analysis, it is concluded that, in general, the problem has one attitude solution. Nonetheless, there are configurations with two or infinite solutions, which are identified in this work.

## 1. Introduction

Attitude determination is the computation of the angular relation between different frames in a given moment. Information about attitude serves a critical role in autonomous navigation of any system. It has been well studied since the dawn of spacecraft technology, when one of the most recognized methods, the Tri-Axial Attitude Determination (TRIAD) algorithm, was developed [1]. This algorithm constructs two triads of orthonormal reference and observation vectors from two non-collinear vector references and observations, which are related by a unique orthogonal matrix. Shortly after, Wahba formulated an optimization problem combining several measurements into a cost function [2]. This problem has remained central in attitude determination, hence many methods for its solution exist. A widely known and employed solution is the Quaternion Estimator (QUEST) algorithm [3], which makes use of the quaternion representation of a rotation matrix. Moreover, there are more recent approaches, such as the fast linear quaternion attitude estimator (FLAE), which can reduce the computational time while having similar accuracy to other methods, as described in [4]. As the variety of applications for autonomous systems expanded, the interest in vehicle formations has grown, because of their potential to solve complex problems with simpler and cheaper individual systems [5], while being more flexible and more fault tolerant, as a whole, than other systems. Their practical applications are numerous and use all kinds of vehicles. For instance, [6] describes a problem for platoons of underwater vehicles, [7] describes a range of applications with unmanned aerial vehicles, whereas [8] presents a survey with applications, both real and potential, for cooperative systems of vehicles, including scenarios with ground, aerial, and marine vehicles working together.

Attitude determination in the context of formations may be challenging, specially if there are constraints that prevent individual vehicles of measuring their own attitude. The three-vehicle formation considered in [9] is constrained to have all measurements in the same plane. Its attitude determination problem is solved considering first both the inertial positions and relative bearing measurements, then parallel relative bearing measurements, and finally non-parallel relative bearing and range measurements. An application of this problem is found in [10], which considers a formation of small satellites. A similar scenario with a coplanar constraint is considered in [11], where the attitude determination problem is solved for a two-vehicle formation, while taking advantage of the observation of a common landmark. A recent attitude problem was addressed in [12], where both hand-eye and vector measurements were used to minimize a cost function and determine the relative attitude between two spacecraft. This work considers the framework laid out in [13], that is, a three-vehicle formation with both line-of-sight (LOS) measurements, relative to other vehicles, and inertial reference measurements, where the LOS observations between two of the vehicles are restricted.

This work is motivated by the lack of a complete analysis on the number of solutions for each measurement set in the attitude determination problem described in [13]. Such analysis is necessary because undesired multiple solutions can substantially affect the estimation errors. Consequently, the identification of these cases is essential for any attitude observer design applied to this specific problem, since such design must handle the potential errors associated with these cases. Otherwise, the existence of multiple solutions may cause the deviation of the estimation errors into intolerable values.

This characterization should provide a way to obtain the number of solutions for the problem without actually applying the solution. A similar analysis for two robots is shown in [14], where a wide range of minimal problems with two vehicles is characterized relative to the number of poses which solve such problems. Nonetheless, a third vehicle introduces more complexity, specifically at the level of information symmetry, which is not considered in that work.

Therefore, the main goal of this paper is to identify the number of attitude solutions of each specific set of measurements, also called a configuration of the formation, considering the framework described in [13]. Moreover, it is interesting to geometrically interpret the special configurations with multiple attitude solutions, since they provide intuition for the problem.

In this work, vision sensors are considered, although alternatives exist. Such sensors provide an accessible and reliable method to extract observations in formations, due to the variety of such sensors available and the independence from outside systems such as the Global Positioning System (GPS). Nonetheless, due to the constraints considered in this problem, the measurement of an inertial reference by each vehicle is required. This requirement allows the use of magnetometers, instead of star trackers, which are a more expensive option and generally already provide a complete inertial attitude estimate.

This formation is denominated as heterogeneous [5] because the sets of sensors, or measurements, for each vehicle are different. As an example, one vehicle has two LOS sensors measuring the relative direction to other vehicles, while the remainder only have one. The potential applications of such a formation is, for example, in multi-spacecraft observatories, in orbit far from Earth, which synthesize large aperture telescopes or long baseline interferometers [15], or even sample spatially disperse phenomena such as the Earth’s magnetotail [16]. In such context, it is desirable to have a large distance between each element of the system, because it improves resolution. Another scenario where this framework can be applied is in chained leader–follower constellations, where each spacecraft only has line of sight relative to its nearest neighbor. In all these, the advantage of the formation in [13] is that it does not require line of sight measurements between the furthermost vehicles to solve the attitude determination problem.

The contributions of this work include the full characterization of all the configurations for which there is more than one set of attitudes that satisfy all the constraints imposed by the measurements. In addition, an intuitive geometric reasoning is given for the different cases. Hence, this paper improves the special configurations’ description in [17], which was based on the analysis of the solution equations. The new results not only provide a full insight to the geometry of the problem, but also include a proof for each of the conclusions. First, the analysis focus on just two of the vehicles of the formation. Their relative and inertial attitudes are shown to have either one, two, or infinite solutions and the specific conditions for each outcome are specified. The cases with a unique solution are related to the problems in [9,11], whereas the cases with infinite solutions correspond to a loss of information in the measurement set. Then, the whole formation is shown to have either one, two, or infinite solutions. Again, the respective configurations are specified. Moreover, the cases with two solutions result from information symmetries and, as before, infinite solutions relate to information loss. Finally, the ambiguous configurations’ subset is shown to be a zero-measure subset, while the simulations show that the neighborhood of such cases is important as well, due to possible estimation errors.

This paper is organized as follows. First, recurrent notation, definitions and properties are described. Then, in Section 3, the problem and respective solution, from [13], are summarized. Section 4 initially characterizes the number of solutions for the relative and inertial attitudes with the measurements from two of the vehicles. Then, it analyzes the number of solutions for the entire formation. The distinction between the general case and some ambiguous configurations requires the analysis of the symmetry of the whole formation, which provides an extra condition for the existence of two solutions. At the end of the section, the geometric intuition for such cases is shown, followed by the proof that these ambiguous configurations are a zero measure subset. Section 5 shows a simulation of a simple maneuver which illustrates and validates the conclusions in this paper. Finally, Section 6 summarizes and comments the results obtained in this work.

## 2. Notation, Definitions, and Properties

Throughout this document, scalars are represented in regular typeface, whereas vectors and matrices are represented in bold, with the latter in capital case. Reference frames are represented in calligraphic typeface and between brackets, such as {I}. Body-fixed frames are numbered and represented by the letter B, with the respective number as a subscript. The symbol 0 represents the null vector or matrix and I represents the identity matrix with the appropriate dimensions. The four-quadrant inverse tangent function is denoted by $\mathrm{atan}2\left(b,a\right)$, with a,b∈R. The set of unit vectors in $R^{3}$ is denoted by $S\left(2\right):=\left\{x\in R^{3}:∥x∥=1\right\}$. The special orthogonal group of dimension 3, which describes proper rotations, is denoted by $\mathrm{SO}\left(3\right):=\left\{X\in R^{3\times 3}:{\mathrm{XX}}^{T}=X^{T}X=I\;\wedge \;\det\left(X\right)=1\right\}$. The skew-symmetric matrix parameterized by x∈$R^{3}$, which encodes the cross product between x and another vector, is denoted by

(1) $$S\left(x\right):=\left[\;\begin{array}{ccc} 0 & -x_{3} & x_{2}\\ x_{3} & 0 & -x_{1}\\ -x_{2} & x_{1} & 0 \end{array}\;\right],\;$$

with $x={\left[x_{1}\;x_{2}\;x_{3}\right]}^{T}$.

The rotation matrix in SO(3) that transforms a given vector, in $R^{3}$, expressed in $\left\{B_{i}\right\}$ to $\left\{B_{j}\right\}$, $i,j\in N_{0}$, is denoted by $R_{i}^{j}$. If a frame is not body-fixed, the respective letter is used instead. Moreover, multiple candidates for the same quantity are identified by a subscript capital case letter, such as ${\left(R_{i}^{j}\right)}_{A}$. The rotation matrix of an angle θ∈R about the axis described by the unit vector x∈S(2) is denoted by R(θ,x), which is written, recalling (1), as [18]

(2) $$R\left(\theta ,x\right):=\cos\left(\theta \right)I+\left(1-\cos\left(\theta \right)\right)x\;x^{T}-\sin\left(\theta \right)S\left(x\right).$$

The following properties are used recurrently throughout the paper. Let θ∈R, x∈S(2), $y\in R^{3}$, A∈SO(3), and $R\left(\theta ,x\right)\in \mathrm{SO}\left(3\right)$. Then,

(3) $$∥R\left(\theta ,x\right)y∥=∥y∥,$$

(4) $$R\left(\theta ,x\right)x=x,$$

(5) $$S\left(\mathrm{Ry}\right)=RS\left(y\right)R^{T},$$

(6) $$R\left(\theta ,\mathrm{Ax}\right)=AR\left(\theta ,x\right)A^{T},$$

and

(7) $$S\left(x\right)R\left(\theta ,x\right)=R\left(\theta ,x\right)S\left(x\right).$$

## 3. Problem and Solution

### 3.1. Problem Definition

Consider a formation with three vehicles, where $\left\{B_{1}\right\}$, $\left\{B_{2}\right\}$, and $\left\{B_{3}\right\}$ are the body-fixed frames of the respective vehicles and {I} represents the inertial frame. In the proposed framework, there are two kinds of measurements: one is a LOS vector that points to the position of another vehicle and the other is an inertial reference vector, for example, a known physical field direction. All measurements are unit vectors obtained in the respective body-fixed frame. Moreover, the inertial references are known in the inertial frame.

**Figure 1:**
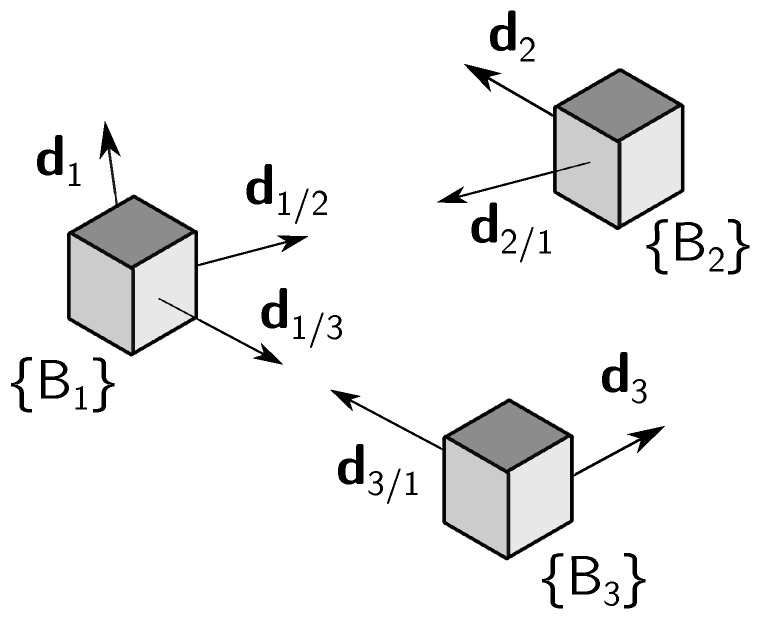
Three-vehicle heterogeneous formation.

In the formation, the main constraint is that two of the vehicles, called the deputies, cannot measure LOS vectors between them. Meaning, for example, that these two vehicles are too far from each other. Furthermore, each vehicle can measure one inertial vector independently. The vehicle that measures LOS to the other two is identified as vehicle 1 and is denominated as chief, whereas the deputies are identified as vehicles 2 and 3. The subgroup with the chief and a deputy is called a branch of the formation, hence there are two branches in this case. The branch 1–2 includes the chief and vehicle 2, whereas branch 1–3 includes the chief and vehicle 3. The geometry of the framework is represented in Figure 1.

In the figure and throughout this document, $d_{i/j}$, i,j=1,2,3, i≠j, represents the LOS vector from the *i*-th to the *j*-th vehicles, expressed in $\left\{B_{i}\right\}$, and $d_{i}$, i=1,2,3, represents the inertial vector measured by the *i*-th platform, expressed in $\left\{B_{i}\right\}$. A left superscript specifying the frame is used whenever a vector is expressed in a different frame. For example, $\;{\;}^{I}\;d_{i}$, i=1,2,3, is the inertial vector of the *i*-th vehicle, expressed in $\left\{I_{}\right\}$.

The problem that is here considered is that of determining all the rotation matrices, both relative ($R_{2}^{1}$, $R_{3}^{1}$, $R_{3}^{2}$) and inertial ($R_{1}^{I}$, $R_{2}^{I}$, $R_{3}^{I}$), using the measurement vectors that were described, as well as the references $\;\;{\;}^{I}\;d_{1}$, $\;{\;}^{I}\;d_{2}$, and $\;{\;}^{I}\;d_{3}$.

### 3.2. Solution

**Figure 2:**
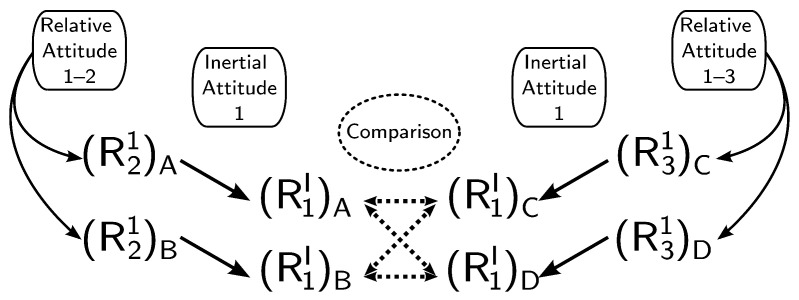
Disambiguation procedure.

The solution proposed in [13] resorts to multiple stages, as represented in Figure 2. First, the candidates for $R_{2}^{1}$ and $R_{3}^{1}$ are determined. Afterwards, these are used to obtain the candidates for $R_{1}^{I}$. Since computing $R_{1}^{I}$ using either $R_{2}^{1}$ or $R_{3}^{1}$ is equivalent, then it is possible to disambiguate the problem. Therefore, a comparison between the candidates for $R_{1}^{I}$ is carried out. Finally, the remaining matrices are found from the disambiguated solutions for $R_{2}^{1}$, $R_{3}^{1}$, and $R_{1}^{I}$. A more detailed algorithm flowchart can be found in [13].

#### 3.2.1. Relative Attitude

In this section, the solution for the candidates of $R_{2}^{1}$ is provided. The solution for $R_{3}^{1}$ is omitted because it is completely analogous. The ensuing derivation also relates to the work in [11], where it is shown that using a planar constraint leads to an ambiguity. In this case, however, the problem is not constrained to a triangle, and therefore, such ambiguity cannot be resolved without extra information, which will be provided when combining the information of both branches. Hence, recall the parameterization (2) and consider the decomposition of the relative attitude given by

(8) $$R_{2}^{1}:=R\left(\theta_{2},n_{2}\right)R\left(\theta_{1},n_{1}\right),$$

with $\theta_{1},\theta_{2}\in R$ and $n_{1},n_{2}\in S\left(2\right)$, such that $R_{2}^{1}$ verifies the constraints expressed as

(9a) $$-d_{1/2}=R_{2}^{1}d_{2/1}$$

and

(9b) $$d_{1}^{T}R_{2}^{1}d_{2}=\;{\;}^{I}\;d_{1}^{T}\;\;\;{\;}^{I}\;d_{2}.$$

The resulting parameters are given by

(10a) $$\theta_{1}:=\pi ,$$

(10b) $$\left\{\begin{array}{cc} n_{1}:=\frac{d_{2/1}-d_{1/2}}{∥d_{2/1}-d_{1/2}∥},\; & \;\mathrm{for}\;d_{2/1}\neq d_{1/2}\\ n_{1}:=\frac{S\left(d_{1/2}\right)d_{1}}{∥S\left(d_{1/2}\right)d_{1}∥},\; & \;\mathrm{for}\;d_{2/1}=d_{1/2} \end{array}\right.,$$

(11a) $$\theta_{2}:=\mathrm{atan}2\left(c_{s_{12}},c_{c_{12}}\right)\pm \arccos\left(\frac{c_{p_{12}}}{\sqrt{c_{s_{12}}^{2}+c_{c_{12}}^{2}}}\right),$$

and

(11b) $$n_{2}:=-d_{1/2},$$

with

(12) $$\left\{\begin{array}{cc} c_{p_{12}} & :=d_{1}^{T}\left(d_{1/2}\right){\left(d_{1/2}\right)}^{T}d_{2}^{🟉}-\;{\;}^{I}\;d_{1}^{T}\;\;{\;}^{I}\;d_{2}\\ c_{c_{12}} & :=d_{1}^{T}S{\left(d_{1/2}\right)}^{2}d_{2}^{🟉}\\ c_{s_{12}} & :=d_{1}^{T}S\left(-d_{1/2}\right)d_{2}^{🟉} \end{array}\right.,$$

where

(13) $$d_{2}^{🟉}:=R\left(\theta_{1},n_{1}\right)d_{2}.$$

As evidenced in (11a), there are, in general, two candidates for $R_{2}^{1}$. Nonetheless, it is possible to have a unique solution when $\theta_{2}$ is unique. The same reasoning applies to $R_{3}^{1}$.

#### 3.2.2. Inertial Attitude

The candidates for $R_{1}^{I}$ are computed using the TRIAD algorithm [1], which considers the relations between the inertial vectors expressed in different frames, as given by

(14a) $$\;{\;}^{I}\;d_{1}=R_{1}^{I}d_{1},$$

(14b) $$\;{\;}^{I}\;d_{2}=R_{1}^{I}R_{2}^{1}d_{2},$$

and

(14c) $$\;{\;}^{I}\;d_{3}=R_{1}^{I}R_{3}^{1}d_{3}.$$

However, for the characterization of the problem, it is useful to describe the candidates for $R_{1}^{I}$ as a product between two rotations, similarly to $R_{2}^{1}$ and $R_{3}^{1}$. For that matter, consider the following alternative method for the determination of $R_{1}^{I}$. Decompose the rotation matrix into a product between two separate rotations, as given by

(15) $$R_{1}^{I}=R\left(\theta_{X_{i}},n_{X}\right)R_{}^{*},$$

with $\theta_{X_{i}}\in R$, $n_{X}\in S\left(2\right)$, and $R_{}^{*}\in \mathrm{SO}\left(3\right)$. The rotation $R_{}^{*}$ verifies (14a) and is given by

(16) $$R_{}^{*}:=\left\{\begin{array}{cc} R\left(\pi ,\frac{\;{\;}^{I}\;d_{1}+d_{1}}{∥\;{\;}^{I}\;d_{1}+d_{1}∥}\right),\; & \;\;{\;}^{I}\;d_{1}\neq -d_{1}\\ R\left(\pi ,\frac{S\left(\;{\;}^{I}\;d_{1}\right)\;{\;}^{I}\;d_{2}}{∥S\left(\;{\;}^{I}\;d_{1}\right)\;{\;}^{I}\;d_{2}∥}\right),\; & \;\;{\;}^{I}\;d_{1}=-d_{1} \end{array}\right..$$

Since (15) verifies (14b) or (14c), according to which branch is being considered, then $n_{X}=\;{\;}^{I}\;d_{1}$. Next, define

(17a) θX2:=atan2Id2TSId1R∗R21X2d2,Id2TSId12R∗R21X2d2+π,

and

(17b) $$\theta_{X_{3}}:=\mathrm{atan}2\left(\;\;{\;}^{I}\;d_{3}^{T}S\left(\;{\;}^{I}\;d_{1}\right)R_{}^{*}{\left(R_{3}^{1}\right)}_{X_{3}}d_{3},\;{\;}^{I}\;d_{3}^{T}S{\left(\;{\;}^{I}\;d_{1}\right)}^{2}R_{}^{*}{\left(R_{3}^{1}\right)}_{X_{3}}d_{3}\right)+\pi ,$$

where $X_{2}$ is substituted by the candidate reference A or B, and $X_{3}$ is substituted by the candidate reference C or D. In conclusion, the inertial attitude candidate for $R_{1}^{I}$ is given by ${\left(R_{1}^{I}\right)}_{X_{i}}=R\left(\theta_{X_{i}},\;{\;}^{I}\;d_{1}\right)R_{}^{*}$, where $X_{i}$ represents the candidate reference A, B, C, or D, accordingly, and $\theta_{X_{i}}$ is defined by the corresponding expression of (17). For example, consider candidate A. Then, ${\left(R_{1}^{I}\right)}_{A}=R\left(\theta_{A},\;{\;}^{I}\;d_{1}\right)R_{}^{*}$, with $\theta_{A}$ obtained from substituting the relative attitude candidate ${\left(R_{2}^{1}\right)}_{A}$ in (17a). Since these results are analogous to the relative attitude solution in [13], the proof is omitted.

#### 3.2.3. Comparison

To compare the candidates for $R_{1}^{I}$, from different branches, consider the parameter defined by

(18) $$\phi_{XY}:=\left|\mathrm{trace}\left[I\right]-\mathrm{trace}\left[{\left(R_{1}^{I}\right)}_{X}{\left(R_{1}^{I}\right)}_{Y}^{T}\right]\right|,$$

where ${\left(R_{1}^{I}\right)}_{X}$ and ${\left(R_{1}^{I}\right)}_{Y}$ represent two different candidates. This parameter is based on the dependence of the rotation matrix trace and its angle of rotation. Thus, ϕ is zero when both candidates are equal, which enables the disambiguation.

#### 3.2.4. Complete Solution

The remaining attitude matrices, that is $R_{3}^{2}$, $R_{2}^{I}$, and $R_{3}^{I}$, are obtained from a product between the attitudes already determined, see [13] for further details.

#### 3.2.5. Sensor Errors

In the presence of sensor errors, the comparison between the inertial candidates is imperfect, hence the values of ϕ are, in general, different from zero. In that case, the solution is given by the smallest ϕ. Optionally, the pair of candidates with the lowest value of ϕ are averaged using the singular value decomposition (SVD) to obtain an improved estimate for $R_{1}^{I}$. Nonetheless, if there are multiple values of ϕ close to zero, the minimum may not correspond to the correct solution. The characterization of the problem given in this work identifies the configurations where such situation may happen.

#### 3.2.6. Computational Complexity

This algorithm is based on a small number of measurements and their operations are relatively simple. Computationally, the operation which is more costly is the SVD that is performed to find a mean rotation, however this operation is optional. Moreover, there are computationally efficient algorithms to perform the SVD of 3 × 3 matrices.

## 4. Characterization

This section analyzes the relation between each possible configuration of the formation and the respective number of attitude solutions. If there is no other information, any attitude set that satisfies all the constraints is considered a solution for the problem. Therefore, it is important to understand which configurations result in more than one solution, otherwise there may be large errors, or even divergence from the correct attitude, in the design of estimators. Thus, these configurations are properly identified in the sequel.

In general, there is only one attitude set which satisfies the constraints for a given configuration. However, there are degenerate configurations, where there are infinitely many attitudes which verify all the constraints. Furthermore, there are ambiguous configurations, where there are exactly two possible attitude sets that satisfy all the constraints.

The degenerate configurations are generated by a set of constraints with an incomplete amount of information, which result in, at least, an extra degree of freedom for the system. Likewise, some ambiguous configurations result from a measurement set with poor quality. Nonetheless, these configurations are usually the result of symmetric information in both branches of the formation, which in turn results in identical pairs of candidates for $R_{1}^{I}$.

The analysis is divided into three parts. First, a branch analysis investigates the constraints used in the branch 1–2 to find the solutions of $R_{2}^{1}$ and $R_{1}^{I}$. Then, it identifies the conditions which lead to degenerate, ambiguous, and unambiguous branches, respectively, with infinite, two, or one solution. Analogous conclusions are taken relative to the branch 1–3. Afterwards, a formation analysis investigates the various conditions found in the branch analysis and connects them to the number of solutions for the entire formation. Finally, a symmetry analysis is required to find ambiguous configurations which are not determined by the number of solutions of the branches. This analysis explores the problem symmetry to find conditions for the ambiguous configurations. Such configurations are explained geometrically and the respective set is shown to be a zero measure subset of all possible configurations. Lastly, the results of this section are discussed and compared with related work.

### 4.1. Branch Analysis

The analysis of a single branch, which includes only two vehicles, hints at the possible number of solutions for the overall formation. The number of candidates in a branch is either one, two, or infinite. Therefore, a branch can be classified as either unambiguous, ambiguous, or degenerate, respectively. Consider the solutions for $R_{2}^{1}$ and $R_{1}^{I}$ using measurements from branch 1–2. The ensuing results and conclusions are analogously applicable to the branch 1–3, due to the symmetry of the problem.

First, consider the solution for $R_{2}^{1}$ given by the parameters from (10) and (11). Since this solution relies on the constraints given in (9a) and (9b), then the degenerate and unambiguous branch configurations are a result of how much information is encoded in these constraints.

The basic idea behind the different number of solutions is that having collinear independent measurements translates into information loss, because one of the axes is not constrained. Moreover, in this case, the coplanarity of the independent vectors eliminates the inherent ambiguity of the branch. In (9a) and (9b), there are three independent vectors, hence two collinear relations are possible: $d_{1}=\pm d_{1/2}$ or $d_{2}=\pm d_{2/1}$.

The effects of each of these conditions are explored before showing the different number of solutions for $R_{2}^{1}$. Assume that the former relation, i.e., $d_{1}=\pm d_{1/2}$, is true. It follows, from (8), (10) and (11), that the constraint (9b) can be rewritten as

$$\;{\;}^{I}\;d_{1}^{T}\;\;{\;}^{I}\;d_{2}=d_{1}^{T}R_{2}^{1}d_{2}=d_{1}^{T}R\left(\theta_{2},-d_{1/2}\right)R\left(\theta_{1},n_{1}\right)d_{2}=d_{1}^{T}R\left(\theta_{2},\mp d_{1}\right)R\left(\theta_{1},n_{1}\right)d_{2},$$

or, equivalently, from (4),

$$\;{\;}^{I}\;d_{1}^{T}\;\;{\;}^{I}\;d_{2}=d_{1}^{T}R\left(\theta_{1},n_{1}\right)d_{2}.$$

Therefore, $R_{2}^{1}$, as defined in (8), satisfies both (9a) and (9b), with any $\theta_{2}\in R$. Moreover, recall (15), (16) and (17a). Since, by definition, $\;{\;}^{I}\;d_{1}=R_{}^{*}d_{1}$, then (6) implies that

(19) $$R_{1}^{I}=R\left(\theta_{X_{2}},\;{\;}^{I}\;d_{1}\right)R_{}^{*}=R_{}^{*}R\left(\theta_{X_{2}},d_{1}\right).$$

Then, substituting (8) and (19) in the constraint (14b) yields

$$\;{\;}^{I}\;d_{2}=R_{1}^{I}R_{2}^{1}d_{2}=R_{}^{*}R\left(\theta_{X_{2}},d_{1}\right)R\left(\theta_{2},-d_{1/2}\right)R\left(\theta_{1},n_{1}\right)d_{2}$$

which, recalling the assumption $d_{1}=\pm d_{1/2}$, can be written as

(20) $$\;{\;}^{I}\;d_{2}=R_{}^{*}R\left(\theta_{X_{2}},d_{1}\right)R\left(\theta_{2},\mp d_{1}\right)R\left(\theta_{1},n_{1}\right)d_{2}=R_{}^{*}R\left(\theta_{X_{2}}\mp \theta_{2},d_{1}\right)R\left(\theta_{1},n_{1}\right)d_{2}.$$

It follows that, $R_{1}^{I}$, as expressed in (19), satisfies both (14a) and (14b) with any $\theta_{X_{2}}\in R$, because $\theta_{2}$ is arbitrary.

Assume instead that $d_{2}=\pm d_{2/1}$. Then, recalling (9a), the constraint (9b) can be rewritten as

(21) $$\;{\;}^{I}\;d_{1}^{T}\;\;{\;}^{I}\;d_{2}=d_{1}^{T}R_{2}^{1}d_{2}=\pm d_{1}^{T}R_{2}^{1}d_{2/1}=\mp d_{1}^{T}d_{1/2}.$$

Consequently, since $R\left(\theta_{1},n_{1}\right)$ satisfies (9a), which can be verified by substituting $R_{2}^{1}$ by $R\left(\theta_{1},n_{1}\right)$ in (9a), it follows that $R_{2}^{1}$, as expressed in (8), satisfies both (9a) and (9b) with any $\theta_{2}\in R$. Additionally, substituting (8) in (14b) gives

$$\;{\;}^{I}\;d_{2}=R_{1}^{I}R_{2}^{1}d_{2}=R_{1}^{I}R\left(\theta_{2},-d_{1/2}\right)R\left(\theta_{1},n_{1}\right)d_{2}.$$

Since $R\left(\theta_{1},n_{1}\right)$ satisfies (9a), then applying the assumption $d_{2}=\pm d_{2/1}$ gives

$$\;{\;}^{I}\;d_{2}=\pm R_{1}^{I}R\left(\theta_{2},-d_{1/2}\right)R\left(\theta_{1},n_{1}\right)d_{2/1}=\mp R_{1}^{I}R\left(\theta_{2},-d_{1/2}\right)d_{1/2},$$

which, from (4), results in

(22) $$\;{\;}^{I}\;d_{2}=\mp R_{1}^{I}d_{1/2}.$$

Hence, if $d_{1}\neq \pm d_{1/2}$ and $\;\;{\;}^{I}\;d_{1}\neq \pm \;{\;}^{I}\;d_{2}$, there is a unique value for $R_{1}^{I}$ that satisfies both (14a) and (14b), because the second constraint becomes independent of $R_{2}^{1}$.

The intuition given before provides some conditions which affect the information encoded in the constraints. However, the connection between the number of solutions and a set of conditions for the measurements relies on the expressions used for the solution. Therefore, the next lemma addresses the relation between the collinear conditions, considered above, and particular values of the coefficients $c_{s_{12}}$ and $c_{c_{12}}$, as defined in (12), which are related to special cases of the solution. It establishes the logical connection used to relate the degenerate cases to an infinite number of solutions for $R_{2}^{1}$. Since this is a technical result, the respective proof is given in Appendix B.

**Lemma** **1.** *Consider $c_{s_{12}}$ and $c_{c_{12}}$ as defined in *(12)*. Then, both $c_{s_{12}}=0$ and $c_{c_{12}}=0$ if and only if either $d_{1}=\pm d_{1/2}$ or $d_{2}=\pm d_{2/1}$.*

Knowing the conditions associated with $c_{s_{12}}=0$ and $c_{c_{12}}=0$ allows to find the degenerate configurations, because that is the only case for which the expressions that result in ${\left(R_{2}^{1}\right)}_{A}$ and ${\left(R_{2}^{1}\right)}_{B}$ are not defined. Furthermore, from (8), with the respective parameters given in (10) and (11), it follows that the ambiguity, which yields two candidates, is encoded in the angle $\theta_{2}$, as is evidenced in (11a). Inspecting such expression leads to the conclusion that there are cases with only one solution for $\theta_{2}$, namely, when

(23) $$\arccos\left(\frac{c_{p_{12}}}{\sqrt{c_{s_{12}}^{2}+c_{c_{12}}^{2}}}\right)=\pi k,$$

with k∈Z.

The next theorem addresses all possible numbers of solutions for $R_{2}^{1}$, considering the measurements of branch 1–2.

**Theorem** **1.** *Consider the relative attitude between vehicles 1 and 2, i.e., $R_{2}^{1}$. Recall the measurements given as $d_{1}$, $d_{2}$, $d_{1/2}$, and $d_{2/1}$. Then:*

*(i)*  *there are infinite solutions if and only if*
  (24a) $$d_{1}=\pm d_{1/2}\;\;$$
  *or*
  (24b) $$d_{2}=\pm d_{2/1};$$
*(ii)*  *the solution is unique if and only if*
  (25) $$d_{1}^{T}\;S\left(d_{1/2}\right)R_{2}^{1}d_{2}=0,$$
  *while*$d_{1}\neq \pm d_{1/2}$*and*$d_{2}\neq \pm d_{2/1}$;
*(iii)*  *otherwise, there are two solutions.*

**Proof.**  This proof is divided into three parts:

(i) Consider that (24a) or (24b) are verified. Then, [13] (Lemma 3) ensures that there are infinite solutions for $R_{2}^{1}$. Otherwise, consider that neither (24a) nor (24b) are verified. Then, it follows from Lemma 1 that either $c_{s_{12}}\neq 0$ or $c_{c_{12}}\neq 0$, or both. In any case, (8)–(13) are all well-defined and there are, at most, two solutions for $R_{2}^{1}$, i.e., a finite number of solutions. By contra-position, if there are infinite solutions for $R_{2}^{1}$, either (24a) or (24b) must be satisfied, thus completing the first part of the proof.
(ii) Assume that neither (24a) nor (24b) are verified, then $\mathrm{atan}2\left(c_{s_{12}},c_{c_{12}}\right)$ is always defined. Since (2) is periodic relative to the angle, with period 2π, it is concluded, from (11a), that $R_{2}^{1}$ has a unique solution if and only if
  (26) $$\theta_{2}=\mathrm{atan}2\left(c_{s_{12}},c_{c_{12}}\right)+\pi k\;,\;k\in Z\;.$$
  It follows that (26) is equivalent to $c_{s_{12}}\cos\left(\theta_{2}\right)=c_{c_{12}}\sin\left(\theta_{2}\right)$. Then, expanding the coefficients by applying (12) and introducing
  $$\left(1-\cos\left(\theta_{2}\right)\right)d_{1}^{T}S\left(-d_{1/2}\right)d_{1/2}d_{1/2}^{T}d_{2}^{🟉}=0$$
  gives
  (27) $$d_{1}^{T}\;S\left(-d_{1/2}\right)\left[\cos\left(\theta_{2}\right)I+\left(1-\cos\left(\theta_{2}\right)\right)d_{1/2}d_{1/2}^{T}-\sin\left(\theta_{2}\right)S\left(-d_{1/2}\right)\right]d_{2}^{🟉}=0.$$
  Recall (2) and (13). Then, (27) is equivalent to (25).
(iii) All other cases result in two solutions for $R_{2}^{1}$, because they yield two distinct values for $\theta_{2}$, considering their representation in the same 2π interval, as concluded from the inspection of (11a). Hence, the proof is complete.

 □

**Remark** **1.** *In short, the first condition, for infinite solutions, is verified when the measurements of one vehicle are collinear. Moreover, there is a unique solution if the involved vectors, given in the same reference frame, are coplanar, but not collinear as in the first case. Otherwise there are two solutions.*

The problems described in [9,11] consider coplanar vectors, which means that (23) is satisfied. Thus, those problems have only one solution in general. Note that the case with a coplanar vector configuration is a particular case of the general problem addressed in the relative attitude solution of the branch. Thus, the information of a second branch is required to disambiguate the solutions.

Finally, consider the solution for $R_{1}^{I}$, with the information available within the branch 1–2. The constraints (14a) and (14b) involve only two independent vectors, and therefore, there is only one possible collinearity condition, which is given by $\;{\;}^{I}\;d_{1}=\pm \;{\;}^{I}\;d_{2}\;.$ Applying such condition in (14b) yields

$$\pm \;\;{\;}^{I}\;d_{1}=R_{}^{*}R\left(\theta_{X_{2}},d_{1}\right)R_{2}^{1}d_{2}$$

or, equivalently,

(28) $$\pm d_{1}=R_{2}^{1}d_{2}.$$

Therefore, $R_{1}^{I}=R_{}^{*}R\left(\theta_{X_{2}},d_{1}\right)$ satisfies (14b) for any $\theta_{X_{2}}\in R$. Furthermore, this condition influences the solution of $R_{2}^{1}$, because it implies that (25) is satisfied. Therefore, if none of the expressions in (24) are verified, there is an unambiguous solution for $R_{2}^{1}$.

As before, to evaluate the number of solutions for $R_{1}^{I}$, the expressions which give such attitude must be considered. Hence, recall that (15), with (16) and (17a) result in candidates ${\left(R_{1}^{I}\right)}_{A}$ and ${\left(R_{1}^{I}\right)}_{B}$, considering candidates ${\left(R_{2}^{1}\right)}_{A}$ and ${\left(R_{2}^{1}\right)}_{B}$. Since the inertial candidates are obtained from the application of the same method used to find the candidates for $R_{2}^{1}$, then the degenerate configurations can be found analogously. Hence, considering the analogous of Lemma 1 and Theorem 1, leads to the conclusion that there are infinite solutions for $R_{1}^{I}$, in the branch 1–2, if and only if $d_{1}=\pm R_{2}^{1}d_{2}$ or $\;{\;}^{I}\;d_{1}=\pm \;{\;}^{I}\;d_{2}.$ In conclusion, since both conditions are equivalent, then there are infinite solutions for $R_{1}^{I}$ if and only if $\;{\;}^{I}\;d_{1}=\pm \;{\;}^{I}\;d_{2}$. In every other case, each relative attitude candidate generates an inertial attitude candidate, because the two independent vectors involved are coplanar by definition, and therefore, satisfy the analogous of (25). The unambiguous character of this solution is expressed by (15)–(17). In such conditions, the number of inertial candidates is the same as the number of relative attitude candidates.

### 4.2. Formation Analysis

The attitude solution for the entire formation results from the fusion of information between both branches. Therefore, the number of attitude solutions of each branch directly influences the number of solutions for the formation.

Recall the nomenclature used to characterize each branch relative to the number of candidates it provides, that is, the branch is degenerate, ambiguous, or unambiguous, respectively, if it has infinite, two, or one solution. Table 1 shows possible combinations of branches configurations and gives the respective number of solutions for the whole formation. Multiple degenerate conditions for each branch can be verified simultaneously, but this analysis, and this table, considers that only one of them, for each branch, is verified at each moment.

#### 4.2.1. Configurations with Infinite Solutions

The conditions which result in infinite solutions for the entire formation require that at least one branch is degenerate, which is expressed in Table 1. All such cases are described below, alongside some details that support the conclusions.

In the cases where both branches are degenerate, all conditions in (14) are satisfied by $R_{1}^{I}=R_{}^{*}R\left(\theta_{X},d_{1}\right)$, with any $\theta_{X}\in R$. Therefore, there are infinite attitude solutions for the problem.

In the case that $d_{2}=\pm d_{2/1}$, (21) and (22) imply that the only constraint which depends on $R_{2}^{1}$ is (9a), and therefore, there are infinite solutions for $R_{2}^{1}$ even if the opposite branch gives a unique candidate for $R_{1}^{I}$ and $R_{3}^{1}$. The same conclusion is analogously applicable to the case with $d_{3}=\pm d_{3/1}$.

#### 4.2.2. Configurations with Two Solutions

The conditions which result in two solutions for the entire formation require that at least one branch is ambiguous, as expressed in Table 1. All such cases are described below, alongside some details that support the conclusions.

Consider the case where one branch is ambiguous and the other is degenerate. First, assume that $d_{1}=\pm d_{1/2}$ while the branch 1–3 is ambiguous. In this case, if $R_{1}^{I}$ is known, then (20) provides a second constraint to $R_{2}^{1}$. Hence, the two different candidates for $R_{1}^{I}$, that emerge from branch 1–3, can be used in (20) to find two candidates for $R_{2}^{1}$. The result is a set of two solutions for the entire formation. Assume instead that $\;{\;}^{I}\;d_{1}=\pm \;{\;}^{I}\;d_{2}$ while the branch 1–3 is ambiguous. Since (28) implies that (25) is satisfied, then there is a unique solution for $R_{2}^{1}$. Therefore, there are two solutions for the formation attitude, because branch 1–3 is ambiguous, which means it gives two candidates for both $R_{1}^{I}$ and $R_{3}^{1}$. The same conclusions arise when considering that branch 1–2 is ambiguous and that either $d_{1}=\pm d_{1/3}$ or $\;{\;}^{I}\;d_{1}=\pm \;{\;}^{I}\;d_{3}$ is satisfied.

According to Table 1, there may be two solutions in the general case where both branches are ambiguous. A more detailed analysis is required to determine these cases. However, the basic idea is that, in such cases, there are two distinct pairs of identical candidates for $R_{1}^{I}$. This analysis follows in Section 4.3, where the conditions which result in such ambiguous cases are found.

#### 4.2.3. Configurations with One Solution

Besides the general case which will be analyzed later, the only other possibility for a unique solution requires that at least one branch is unambiguous.

Consider the case where the branch 1–3 is unambiguous. Hence, there is a unique solution for $R_{3}^{1}$ and $R_{1}^{I}$. The goal of this analysis is to understand whether a single candidate for $R_{2}^{1}$ can be extracted as well. Assume that $d_{1}=\pm d_{1/2}$, then (28) can be used as a constraint for $R_{2}^{1}$, given that $R_{1}^{I}$ is already determined. Therefore, a unique solution for $R_{2}^{1}$ exists. Assume instead that $\;{\;}^{I}\;d_{1}=\pm \;{\;}^{I}\;d_{2}$. Since (25) is verified, then, from Theorem 1, there is a unique solution for $R_{2}^{1}$, and therefore, a unique solution for all attitudes. Otherwise, in the case where one branch is unambiguous and the other is ambiguous and in the case where both branches are unambiguous, there is clearly a unique solution for every attitude of the formation.

### 4.3. Symmetry Analysis

The number of solutions of the branches is not enough to determine the number of solutions of the formation when both branches give two candidates for each attitude. In this case, the symmetry between both branches is responsible for the number of compatible attitudes. The ensuing analysis completes the picture presented in Table 1, by showing whether the solution is unique or ambiguous, when both branches are ambiguous.

An important detail for the ensuing analysis is that any three-dimensional unit vector can be written as the result of two rotations whose axes are orthogonal to each other. Consider that $\;{\;}^{I}\;d_{1}\neq \pm \;{\;}^{I}\;d_{2}$ and $\;{\;}^{I}\;d_{1}\neq \pm \;{\;}^{I}\;d_{1/2}$, which are verified when both branches are ambiguous, then for some combination of angles $\alpha_{1}$, $\alpha_{2}$, $\beta_{1}$, and $\beta_{2}$, it follows that

(29a) Id3=Rα1,Id1Rβ1,SId1Id2∥SId1Id2∥Id2.

and

(29b) $$\;{\;}^{I}\;d_{1/3}=R\left(\alpha_{2},\;{\;}^{I}\;d_{1}\right)R\left(\beta_{2},\frac{S\left(\;{\;}^{I}\;d_{1}\right)\;{\;}^{I}\;d_{1/2}}{∥S\left(\;{\;}^{I}\;d_{1}\right)\;{\;}^{I}\;d_{1/2}∥}\right)\;{\;}^{I}\;d_{1/2}.$$

The notation and definitions, which are given next, simplify the expressions used to determine the ambiguous configurations. Hence, consider the inertial attitude candidates, which, recalling (15), are given as

(30a) $${\left(R_{1}^{I}\right)}_{A}=R\left(\theta_{A},\;{\;}^{I}\;d_{1}\right)R_{}^{*},$$

(30b) $${\left(R_{1}^{I}\right)}_{B}=R\left(\theta_{B},\;{\;}^{I}\;d_{1}\right)R_{}^{*},$$

(30c) $${\left(R_{1}^{I}\right)}_{C}=R\left(\theta_{C},\;{\;}^{I}\;d_{1}\right)R_{}^{*},$$

and

(30d) $${\left(R_{1}^{I}\right)}_{D}=R\left(\theta_{D},\;{\;}^{I}\;d_{1}\right)R_{}^{*},$$

with $R_{}^{*}$ defined in (16) and $\theta_{A}$, $\theta_{B}$, $\theta_{C}$, and $\theta_{D}$ defined in (17). Moreover, denote the reference measurements in the intermediate coordinate frame as

(31a) $$d_{2A}^{*}:=R_{}^{*}{\left(R_{2}^{1}\right)}_{A}d_{2},$$

(31b) $$d_{2B}^{*}:=R_{}^{*}{\left(R_{2}^{1}\right)}_{B}d_{2},$$

(32a) $$d_{3C}^{*}:=R_{}^{*}{\left(R_{3}^{1}\right)}_{C}d_{3},$$

and

(32b) $$d_{3D}^{*}:=R_{}^{*}{\left(R_{3}^{1}\right)}_{D}d_{3}.$$

Therefore applying (30)–(32) to (14b) and (14c), gives

(33a) $$\;{\;}^{I}\;d_{2}=R\left(\theta_{A},\;{\;}^{I}\;d_{1}\right)R_{}^{*}{\left(R_{2}^{1}\right)}_{A}d_{2}=R\left(\theta_{A},\;{\;}^{I}\;d_{1}\right)d_{2A}^{*},$$

(33b) $$\;{\;}^{I}\;d_{2}=R\left(\theta_{B},\;{\;}^{I}\;d_{1}\right)R_{}^{*}{\left(R_{2}^{1}\right)}_{B}d_{2}=R\left(\theta_{B},\;{\;}^{I}\;d_{1}\right)d_{2B}^{*},$$

(34a) $$\;{\;}^{I}\;d_{3}=R\left(\theta_{C},\;{\;}^{I}\;d_{1}\right)R_{}^{*}{\left(R_{3}^{1}\right)}_{C}d_{3}=R\left(\theta_{C},\;{\;}^{I}\;d_{1}\right)d_{3C}^{*},$$

and

(34b) $$\;{\;}^{I}\;d_{3}=R\left(\theta_{D},\;{\;}^{I}\;d_{1}\right)R_{}^{*}{\left(R_{3}^{1}\right)}_{D}d_{3}=R\left(\theta_{D},\;{\;}^{I}\;d_{1}\right)d_{3D}^{*}.$$

Additionally, consider a unit vector a∈S(2) and define the normal to a plane given by $\;{\;}^{I}\;d_{1}$ and a as

(35) $$m_{a}:=\frac{S\left(\;{\;}^{I}\;d_{1}\right)a}{∥S\left(\;{\;}^{I}\;d_{1}\right)a∥}.$$

Finally, consider the superscript ⊥ to denote the transformation which makes a given vector orthogonal to $\;{\;}^{I}\;d_{1}$, as follows

(36) $${\left(a\right)}^{⊥}:=R\left(\psi_{a},m_{a}\right)a$$

with $a\neq \pm \;\;{\;}^{I}\;d_{1}\;$ and $\psi_{a}\in R$, such that

(37) $$\;{\;}^{I}\;d_{1}^{T}{\left(a\right)}^{⊥}=0.$$

#### 4.3.1. Relation between Measurements of the Same Branch

The transformation defined in (36) allows to represent the system measurements in a plane orthogonal to $\;{\;}^{I}\;d_{1}$, which is convenient for establishing the relations between $\;{\;}^{I}\;d_{1/2}$ and $\;{\;}^{I}\;d_{2}$, and analogously between $\;{\;}^{I}\;d_{1/3}$ and $\;{\;}^{I}\;d_{3}$. Such relations are respectively derived in Lemmas A5–A8, in Appendix C, and assume that the candidates that are identical to the inertial attitude are known. Hence, recalling the angles and candidates defined in (30) and assuming that both branches are ambiguous, as defined in Theorem 1, if $R_{1}^{I}={\left(R_{1}^{I}\right)}_{A}$, then

(38a) $${\left(\;{\;}^{I}\;d_{1/2}\right)}^{⊥}=R\left(\frac{\theta_{A}-\theta_{B}}{2}+\pi k_{A},\;{\;}^{I}\;d_{1}\right){\left(\;{\;}^{I}\;d_{2}\right)}^{⊥}.$$

Otherwise, if $R_{1}^{I}={\left(R_{1}^{I}\right)}_{B}$, then

(38b) $${\left(\;{\;}^{I}\;d_{1/2}\right)}^{⊥}=R\left(-\frac{\theta_{A}-\theta_{B}}{2}+\pi k_{B},\;{\;}^{I}\;d_{1}\right){\left(\;{\;}^{I}\;d_{2}\right)}^{⊥}.$$

Moreover, if $R_{1}^{I}={\left(R_{1}^{I}\right)}_{C}$, then

(39a) $${\left(\;{\;}^{I}\;d_{1/3}\right)}^{⊥}=R\left(\frac{\theta_{C}-\theta_{D}}{2}+\pi k_{C},\;{\;}^{I}\;d_{1}\right){\left(\;{\;}^{I}\;d_{3}\right)}^{⊥}.$$

Otherwise, if $R_{1}^{I}={\left(R_{1}^{I}\right)}_{D}$, then

(39b) $${\left(\;{\;}^{I}\;d_{1/3}\right)}^{⊥}=R\left(-\frac{\theta_{C}-\theta_{D}}{2}+\pi k_{D},\;{\;}^{I}\;d_{1}\right){\left(\;{\;}^{I}\;d_{3}\right)}^{⊥},$$

with $k_{A},k_{B},k_{C},k_{D}\in Z$.

#### 4.3.2. Relation between Same Type of Measurement

The relation between measurements across branches completes the set of relations required to establish a connection between the identical candidates and the system measurements.

The only measurement shared by both branches is $\;{\;}^{I}\;d_{1}$, hence the transformation defined in (36) is also convenient for establishing the relation between $\;{\;}^{I}\;d_{2}$ and $\;{\;}^{I}\;d_{3}$, and analogously between $\;{\;}^{I}\;d_{1/2}$ and $\;{\;}^{I}\;d_{1/3}$. Such relations are derived in Lemmas A9 and A10, in Appendix D, and are given as

(40) Id3⊥=Rα1+πk1,Id1Id2⊥,

and

(41) $${\left(\;{\;}^{I}\;d_{1/3}\right)}^{⊥}=R\left(\alpha_{2}+\pi k_{2},\;{\;}^{I}\;d_{1}\right){\left(\;{\;}^{I}\;d_{1/2}\right)}^{⊥},$$

with $\alpha_{1}$ and $\alpha_{2}$ respectively defined in (29a) and (29b), and with $k_{1},k_{2}\in Z$.

#### 4.3.3. Ambiguous Conditions

Advancing to the determination of the ambiguous conditions, recall that the ambiguous configurations with ambiguous branches satisfy either

(42a) $$\left\{\begin{array}{c} {\left(R_{1}^{I}\right)}_{A}={\left(R_{1}^{I}\right)}_{C}\\ {\left(R_{1}^{I}\right)}_{B}={\left(R_{1}^{I}\right)}_{D} \end{array}\right.$$

or

(42b) $$\left\{\begin{array}{c} {\left(R_{1}^{I}\right)}_{A}={\left(R_{1}^{I}\right)}_{D}\\ {\left(R_{1}^{I}\right)}_{B}={\left(R_{1}^{I}\right)}_{C} \end{array}\right.,$$

while both ${\left(R_{1}^{I}\right)}_{A}\neq {\left(R_{1}^{I}\right)}_{B}$ and ${\left(R_{1}^{I}\right)}_{C}\neq {\left(R_{1}^{I}\right)}_{D}$. Thus, the definitions in (30) applied to (42) imply one of two cases:

1. $\theta_{A}=\theta_{C}$ and $\theta_{B}=\theta_{D}$;
2. $\theta_{A}=\theta_{D}$ and $\theta_{B}=\theta_{C}$;

with $\theta_{A}\neq \theta_{B}$ and $\theta_{C}\neq \theta_{D}$.

The main result of this work relates the ambiguous definitions in (42) with the set of formation configurations. For that purpose, it considers the relations encoded in (29) and is given in the following theorem.

**Theorem** **2.** *Consider the definitions for the four candidates of $R_{1}^{I}$ and the angles $\theta_{A}$, $\theta_{B}$, $\theta_{C}$, and $\theta_{D}$ defined in *(30)* and expressed in the same interval of length 2π. Assume that*

(43a) $$\theta_{A}-\theta_{B}\neq 2\pi n$$

*and that*

(43b) $$\theta_{C}-\theta_{D}\neq 2\pi n$$

*with n∈Z. Consider as well the angles $\alpha_{1},\alpha_{2}\in R$ defined in *(29)*. Then,*

(44a) $$\theta_{A}=\theta_{C}\wedge \theta_{B}=\theta_{D}$$

*or*

(44b) $$\theta_{A}=\theta_{D}\wedge \theta_{B}=\theta_{C}$$

*if and only if*

(45) $$\alpha_{1}=\alpha_{2}+\pi k,$$

*with k∈Z.*

**Proof.**  This proof is divided into four parts, one for each possible pair of correct candidates for $R_{1}^{I}$ i.e., one for each of the cases given as follows

(i) $R_{1}^{I}={\left(R_{1}^{I}\right)}_{A}={\left(R_{1}^{I}\right)}_{C}$;
(ii) $R_{1}^{I}={\left(R_{1}^{I}\right)}_{A}={\left(R_{1}^{I}\right)}_{D}$;
(iii) $R_{1}^{I}={\left(R_{1}^{I}\right)}_{B}={\left(R_{1}^{I}\right)}_{C}$;
(iv) $R_{1}^{I}={\left(R_{1}^{I}\right)}_{B}={\left(R_{1}^{I}\right)}_{D}$.

First, consider that (i) is verified. Then, recall (43) and substitute (38a) in (40) which gives

$${\left(\;{\;}^{I}\;d_{3}\right)}^{⊥}=R\left(\alpha_{1}-\frac{\theta_{A}-\theta_{B}}{2}+\pi k,\;{\;}^{I}\;d_{1}\right){\left(\;{\;}^{I}\;d_{1/2}\right)}^{⊥},$$

which from (41) is expressed as

$${\left(\;{\;}^{I}\;d_{3}\right)}^{⊥}=R\left(\alpha_{1}-\alpha_{2}-\frac{\theta_{A}-\theta_{B}}{2}+\pi k,\;{\;}^{I}\;d_{1}\right){\left(\;{\;}^{I}\;d_{1/3}\right)}^{⊥}.$$

Finally, recalling (43) and substituting (39a) results in

(46) $${\left(\;{\;}^{I}\;d_{3}\right)}^{⊥}=R\left(\alpha_{1}-\alpha_{2}+\frac{-\left(\theta_{A}-\theta_{B}\right)+\left(\theta_{C}-\theta_{D}\right)}{2}+\pi k,\;{\;}^{I}\;d_{1}\right){\left(\;{\;}^{I}\;d_{3}\right)}^{⊥}.$$

Recalling (30), then case (i) implies that $\theta_{A}=\theta_{C}$. Therefore, (46) is rewritten as

(47) $${\left(\;{\;}^{I}\;d_{3}\right)}^{⊥}=R\left(\alpha_{1}-\alpha_{2}+\frac{\theta_{B}-\theta_{D}}{2}+\pi k,\;{\;}^{I}\;d_{1}\right){\left(\;{\;}^{I}\;d_{3}\right)}^{⊥}.$$

If (44a) is verified, then $\theta_{B}=\theta_{D}$ and thus (47) implies (45). Conversely, if (45) is verified, then (47) results in (44a). Moreover, since $\theta_{A}=\theta_{C}$, then (44b) is out of the scope of the assumptions in (43), because it would mean that $\theta_{A}=\theta_{B}=\theta_{C}=\theta_{D}$ in the same 2π interval. Hence, the first part of the proof is complete. The remaining cases follow the same train of thought. Consider case (ii), which implies that $\theta_{A}=\theta_{D}$. Taking (40), recalling (43), and substituting (38a), (39b) and (41), and $\theta_{A}=\theta_{D}$, in that order, gives

(48) $${\left(\;{\;}^{I}\;d_{3}\right)}^{⊥}=R\left(\alpha_{1}-\alpha_{2}+\frac{\theta_{B}-\theta_{C}}{2}+\pi k,\;{\;}^{I}\;d_{1}\right){\left(\;{\;}^{I}\;d_{3}\right)}^{⊥}.$$

If (44b) is verified, then $\theta_{B}=\theta_{C}$ and thus (48) implies (45). Conversely, if (45) is verified, then (48) results in (44b). Moreover, since $\theta_{A}=\theta_{D}$, then (44a) is out of the scope of the assumptions in (43), because it would mean that $\theta_{A}=\theta_{B}=\theta_{C}=\theta_{D}$ in the same 2π interval. Hence, the second part of the proof is complete. Next, consider case (iii), which implies that $\theta_{B}=\theta_{C}$. Taking (40), recalling (43), and substituting (38b), (39a) and (41), and $\theta_{B}=\theta_{C}$, in that order, gives

(49) $${\left(\;{\;}^{I}\;d_{3}\right)}^{⊥}=R\left(\alpha_{1}-\alpha_{2}+\frac{\theta_{A}-\theta_{D}}{2}+\pi k,\;{\;}^{I}\;d_{1}\right){\left(\;{\;}^{I}\;d_{3}\right)}^{⊥}.$$

If (44b) is verified, then $\theta_{A}=\theta_{D}$ and thus (49) implies (45). Conversely, if (45) is verified, then (49) results in (44b). Moreover, since $\theta_{B}=\theta_{C}$, then (44a) is out of the scope of the assumptions in (43), because it would mean that $\theta_{A}=\theta_{B}=\theta_{C}=\theta_{D}$ in the same 2π interval. Hence, the third part of the proof is complete. Finally, consider case (iv), which implies that $\theta_{B}=\theta_{D}$. Taking (40), recalling (43), and substituting (38b), (39b) and (41), and $\theta_{B}=\theta_{D}$, in that order, gives

(50) $${\left(\;{\;}^{I}\;d_{3}\right)}^{⊥}=R\left(\alpha_{1}-\alpha_{2}+\frac{\theta_{A}-\theta_{C}}{2}+\pi k,\;{\;}^{I}\;d_{1}\right){\left(\;{\;}^{I}\;d_{3}\right)}^{⊥}.$$

If (44a) is verified, then $\theta_{A}=\theta_{C}$ and thus (50) implies (45). Conversely, if (45) is verified, then (50) results in (44a). Moreover, since $\theta_{B}=\theta_{D}$, then (44b) is out of the scope of the assumptions in (43), because it would mean that $\theta_{A}=\theta_{B}=\theta_{C}=\theta_{D}$ in the same 2π interval, which completes this proof. □

**Remark** **2.** *The configurations where $\theta_{A}=\theta_{B}=\theta_{C}=\theta_{D}$ may satisfy *(45)*. However, such configurations were already defined by Theorem 1 and the conditions which define them do not depend on the angles $\alpha_{1}$ and $\alpha_{2}$. Therefore, the cases where $\theta_{A}=\theta_{B}=\theta_{C}=\theta_{D}$ can be distinguished from the truly ambiguous configurations.*

Importantly, left multiplying (29b) by $R_{1}^{I}$ and recalling (5)–(7), implies that the relations in (29) can be rewritten as

Id3=Rα1,Id1Rβ1,SId1Id2∥SId1Id2∥Id2.

and

$$d_{1/3}=R\left(\alpha_{2},d_{1}\right)R\left(\beta_{2},\frac{S\left(d_{1}\right)d_{1/2}}{∥S\left(d_{1}\right)d_{1/2}∥}\right)d_{1/2}.$$

Therefore, the ambiguous configurations can be determined without solving the attitude problem.

#### 4.3.4. Geometric Interpretation

**Figure 3:**
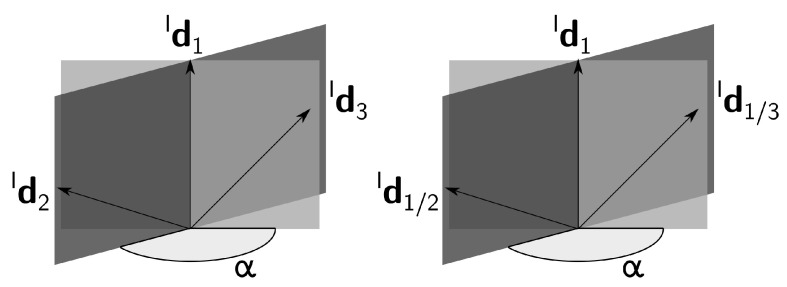
Geometric interpretation of the ambiguous configuration condition with planes.

The application of the constraint given by (45) to the relations in (29) provides a simple geometric interpretation in the inertial frame. Consider the planes defined by $\;{\;}^{I}\;d_{1}$ and each of the other measurements, as represented in Figure 3. If the angle between the planes with LOS measurements (i.e., $\;{\;}^{I}\;d_{1/2}$, $\;{\;}^{I}\;d_{1/3}$) is the same as the angle between the planes with inertial references (i.e., $\;{\;}^{I}\;d_{2}$, $\;{\;}^{I}\;d_{3}$), then the configuration is ambiguous, assuming that the branches are ambiguous as well.

**Figure 4:**
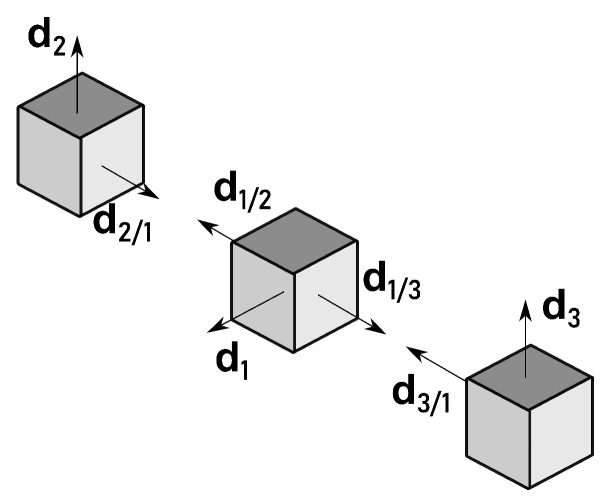
Ambiguous solution: collinear formation.

For another interpretation of this symmetry, take the perspective of the information available for each branch. If the information about the inertial frame is identical in both branches, then an ambiguous solution results from the two ambiguous branches. A simple example is the collinear formation with parallel inertial vectors of the deputies, as represented in Figure 4. In this case, both branches are equivalent in the inertial frame, that is $\alpha_{1}=\beta_{1}=\beta_{2}=0$ and $\alpha_{2}=\pi$. Thus, the two candidates for $R_{1}^{I}$ from both branches coincide. This situation is even more evident when $R_{2}^{1}=R_{3}^{1}$, because both candidates for $R_{2}^{1}$ are identical to both candidates for $R_{3}^{1}$.

The measurements of the configuration in Figure 4 can be transformed according to three degrees of freedom given by $\alpha_{1}$, $\beta_{1}$, and $\beta_{2}$ as defined in (29), with $\alpha_{2}$ constrained by (45). Thus, there are infinite such configurations. Nonetheless, these are a zero measure subset of all possible configurations, as it will be shown next.

#### 4.3.5. Ambiguous Configurations Subset Measure

An interesting and very important property of the subset of ambiguous configurations is its measure. Even though it is shown that the set of ambiguous configurations is, in fact, a zero measure subset, this does not mean that these configurations can be ignored. As it will be shown in the simulations, the ambiguous configurations can have an impact in the attitude estimation when noise is introduced and the formation is near the ambiguity. Hence the importance of this study.

Consider the parameterization of x∈S(2) given by

(51) $$\psi \left(x\right)=\left(\mathrm{atan}2\left(x_{2},x_{1}\right),\arccos\left(x_{3}\right)\right)$$

with $x={\left[x_{1},x_{2},x_{3}\right]}^{T}$. Furthermore, consider the parameterization of X∈SO(3) given by

(52) $$\nu \left(X\right)=\left(\arccos\left(\frac{\mathrm{trace}\left(X\right)-1}{2}\right),\psi \left(\mu_{X}\right)\right)\;,$$

where $\mu_{X}$ is the rotation axis of X.

The following theorem shows that the subset of ambiguous configurations is a zero measure subset of the configuration set.

**Theorem** **3.** *Consider the octuple $\Delta =\left(d_{1/2},d_{1/3},\;{\;}^{I}\;d_{1},\;{\;}^{I}\;d_{2},\;{\;}^{I}\;d_{3},R_{I}^{1},R_{I}^{2},R_{I}^{3}\right)$. Denote the set of all possible configurations of the formation by*

$$M=\left\{\Delta :d_{1/2},d_{1/3},\;{\;}^{I}\;d_{1},\;{\;}^{I}\;d_{2},\;{\;}^{I}\;d_{3}\in S\left(2\right),R_{I}^{1},R_{I}^{2},R_{I}^{3}\in \mathrm{SO}\left(3\right)\right\}\;.$$

*The ambiguous configurations subset is $M_{A}=\left\{\Delta \in M:g_{1}\left(\Delta \right)=0,g_{2}\left(\Delta \right)=0\right\}$, whose functions $g_{1}$ and $g_{2}$ are defined as*

(53a) $$g_{1}\left(\Delta \right)=R\left(\alpha ,R_{I}^{1}\;{\;}^{I}\;d_{1}\right)R\left(\beta ,\frac{S\left(R_{I}^{1}\;\;{\;}^{I}\;d_{1}\right)d_{1/3}}{∥S\left(R_{I}^{1}\;{\;}^{I}\;d_{1}\right)d_{1/3}∥}\right)d_{1/3}-d_{1/2}$$

*and*

(53b) $$g_{2}\left(\Delta \right)\;=\;R\left(\alpha ,\;{\;}^{I}\;d_{1}\right)R\left(\;\gamma ,\frac{S\left(\;{\;}^{I}\;d_{1}\right)\;{\;}^{I}\;d_{3}}{∥S\left(\;{\;}^{I}\;d_{1}\right)\;{\;}^{I}\;d_{3}∥}\;\right)\;\;{\;}^{I}\;d_{3}\;-\;\;{\;}^{I}\;d_{2},$$

*with α,β,γ∈R. Then, $M_{A}$ is a zero measure subset of M.*

**Proof.**  First, note that S(2) [19] (Example 1.2) and SO(3) [20] (Appendix C.2.1) are smooth manifolds, thus *M* is a smooth manifold as well, because it results from the Cartesian product between elements in S(2) and SO(3) [19] (Example 1.13). Furthermore, *M* is a 19-dimensional manifold. Next, consider a collection of subsets covering $M_{A}$ and denoted by $\left\{U_{i}\right\}$, where each $U_{i}$ is small enough to have vectors in S(2) parameterized by both angles of the spherical coordinates, as given by (51), and transformations in SO(3) parameterized by three angles as encoded in (52). Considering $\Delta \in M_{A}$, if all elements but $d_{1/2}$ are known, then $d_{1/2}$ is determined by the angle β, from (53a), alone, because $g_{1}\left(\Delta \right)=0$ and $g_{2}\left(\Delta \right)=0$. Thus, there exists a homeomorphism $\phi_{i}:U_{i}\to {\tilde{U}}_{i}\subset R^{18}$ for every *i* in the collection $\left\{U_{i}\right\}$ and $\left\{\left(U_{i},\phi_{i}\right)\right\}$ is an atlas covering $M_{A}$. Furthermore, every $\phi_{i}\left(U_{i}\cap M_{A}\right)$ has zero Lebesgue measure in $R^{19}$, because these are subsets of $R^{18}$. Therefore, $M_{A}$ is a zero measure subset of *M* [19] (Lemma 6.4). □

### 4.4. Discussion of Results

The branch analysis showed that, in general, there are ambiguities in the branch measurements and therefore two attitudes satisfy the measurement constraints. These ambiguities were identified in [9,11], where the authors constrain their problem geometry such that it gives a unique solution. Theorem 1 identifies the same condition used to ensure the uniqueness of the solution in those works. However, in the formation considered in this work, such constraint is not required, because there is more information available in the other branch. Thus, by combining the information of both branches, a unique solution can still be determined.

The formation analysis considers the fusion of the information in both branches and how the number of solutions of each branch impacts the number of solutions of the entire problem. It was shown that combining the data in both branches reduces the limitations inherent to the isolated branch. This conclusion is expressed in Table 1, where it is visible that the number of solutions for the entire formation can be unique even when there are branches with two or infinite solutions.

The symmetry analysis distinguishes the configurations in which the branch ambiguities extend to the entire formation, from the configurations which have a unique solution. In general, the latter case is verified. In the former case, the ambiguities at the level of the formation result from symmetries between both branches. The simple geometric intuition given by Figure 3 is useful to evaluate whether the configuration is ambiguous or not, and can be employed while designing estimators.

Finally, the fact that all the configurations with multiple solutions are a zero measure subset of the entire configuration space is relevant for the design of estimators, because it means that such designs have the potential to converge to small errors for almost all configurations.

## 5. Simulation

In this section, a simulation illustrates and validates the characterization of the attitude problem considered in this article. Since the degenerate and ambiguous configurations are independent of the attitudes, then, in this simulation and for simplicity, their values are constant and equal to the identity matrix. At the initial time, consider the formation with a unique solution, where the LOS measurements, represented in the inertial frame, are given as $\;\;{\;}^{I}\;d_{1/2}=-\;\;{\;}^{I}\;d_{2/1}={\left[\begin{array}{ccc} \frac{1}{\sqrt{2}} & -\frac{1}{\sqrt{2}} & 0 \end{array}\right]}^{T}$ and $\;\;{\;}^{I}\;d_{1/3}=-\;\;{\;}^{I}\;d_{3/1}={\left[\begin{array}{ccc} 1 & 0 & 0 \end{array}\right]}^{T}$. Furthermore, the inertial references are given as $\;\;{\;}^{I}\;d_{1}={\left[\begin{array}{ccc} 0 & 0 & 1 \end{array}\right]}^{T}$ and $\;\;{\;}^{I}\;d_{2}=\;{\;}^{I}\;d_{3}={\left[\begin{array}{ccc} 0 & 1 & 0 \end{array}\right]}^{T}$. A maneuver of vehicle 2 changes the formation configuration such that the formation reaches an ambiguous configuration and a degenerate configuration at different times. This maneuver consists in varying the position of vehicle 2, which implies a variation of $\;\;{\;}^{I}\;d_{1/2}$ and $\;\;{\;}^{I}\;d_{2/1}$, because $\;\;{\;}^{I}\;d_{1/2}=-\;\;{\;}^{I}\;d_{2/1}$. In total, the maneuver takes 100 s and rotates $\;{\;}^{I}\;d_{1/2}$ by π radians through the axis ${\left[\begin{array}{ccc} 0 & 0 & 1 \end{array}\right]}^{T}$. The remaining vectors and all attitudes are constant throughout the simulation.

Assuming position sensing diodes as sensors, the measurement noise follows the large field of view sensor model [21]. Therefore, the measurement in the sensor frame, ${}^{s}d_{j}$, is related to the focal coordinates $\xi_{j}={\left[\begin{array}{cc} \chi_{j} & \psi_{j} \end{array}\right]}^{T}$, considering a unit focal length, by ${}^{s}d_{j}=\frac{1}{\sqrt{1+\chi_{j}^{2}+\psi_{j}^{2}}}{\left[\begin{array}{ccc} \chi_{j} & \psi_{j} & 1 \end{array}\right]}^{T}$. The focal covariance is given as

(54) $$P_{Fj}=\frac{\sigma_{j}^{2}}{1+\chi_{j}^{2}+\psi_{j}^{2}}\left[\;\begin{array}{cc} cc{\left(1+\chi_{j}^{2}\right)}^{2} & {\left(\chi_{j}\psi_{j}\right)}^{2}\\ {\left(\chi_{j}\psi_{j}\right)}^{2} & {\left(1+\psi_{j}^{2}\right)}^{2} \end{array}\;\right]\;,$$

where $\sigma_{j}$ is a known standard deviation. Then, the covariance of the sensor is given as

(55) $$P_{j}=J_{j}\;P_{Fj}\;J_{j}^{T},$$

where $J_{j}=\frac{\partial {\;}^{s}d_{j}}{\partial \xi_{j}}$ is the respective Jacobian of the relation between the sensor and focal coordinates.

For simplicity, it is assumed that the sensors are aligned with the standard unit vector of the maximum component of the measurement in the body-fixed frame. Thus, the six possible transformations between the body-fixed frame and the sensor frame are given by

$$\left[\begin{array}{ccc} 0 & 0 & -1\\ 0 & 1 & 0\\ 1 & 0 & 0 \end{array}\right],\left[\begin{array}{ccc} 0 & 0 & 1\\ 0 & 1 & 0\\ -1 & 0 & 0 \end{array}\right],\left[\begin{array}{ccc} 1 & 0 & 0\\ 0 & 0 & -1\\ 0 & 1 & 0 \end{array}\right],\left[\begin{array}{ccc} 1 & 0 & 0\\ 0 & 0 & 1\\ 0 & -1 & 0 \end{array}\right],\left[\begin{array}{ccc} 1 & 0 & 0\\ 0 & 1 & 0\\ 0 & 0 & 1 \end{array}\right],\;\mathrm{and}\;\left[\begin{array}{ccc} -1 & 0 & 0\\ 0 & 1 & 0\\ 0 & 0 & -1 \end{array}\right],$$

respectively when the maximum component of the measurement is *x*, −x, *y*, −y, *z*, and −z, where the axis are standard unit vectors of the coordinate frame.

The simulation takes 100 s, the same duration of the maneuver. It is assumed that all sensors are synchronized and have a sampling rate of 10 Hertz, which means that a measurement is taken every 0.1 s. Moreover, all sensors are characterized by $\sigma =17\times 10^{-6}$ radians. At each sampling instant, each measurement is sampled as follows: first, its true value is represented in the respective sensor frame, which, in turn, gives the true focal coordinates. Both are used to compute the sensor covariance, P, by applying (54) and (55). The respective noise is sampled from a zero mean normal distribution with covariance P, which is then added to the measurement. Moreover, the inertial references, in the inertial frame, are known a priori and considered noiseless. With the complete set of measurements, corrupted by noise, the attitude is computed using the algorithm described in Section 3.

**Figure 5:**
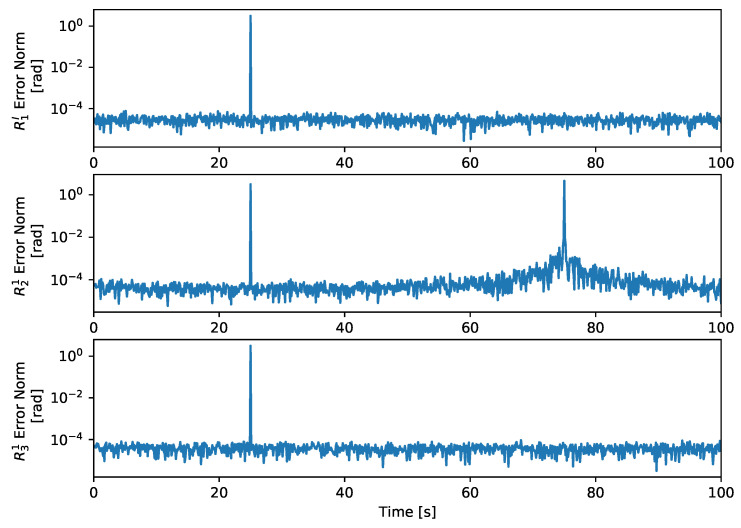
Euler error vector norm.

**Figure 6:**
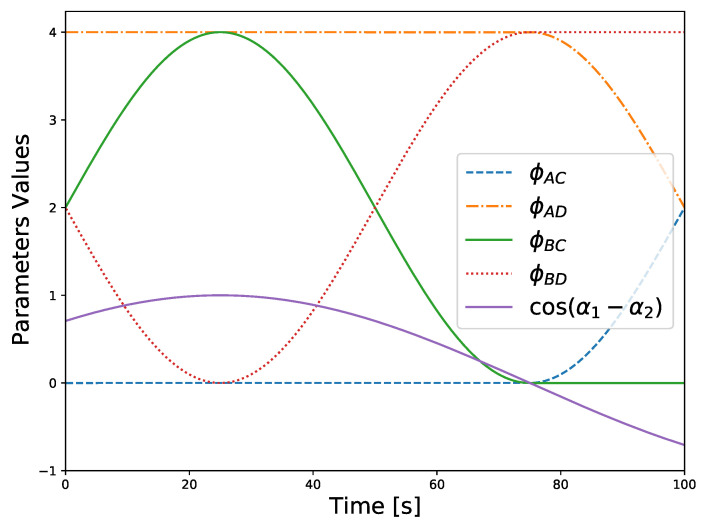
Relation between candidates for $R_{1}^{I}$.

The results of this simulation are given by the estimation errors, the relation between the values of $\theta_{A}$, $\theta_{B}$, $\theta_{C}$, and $\theta_{D}$, and also by the relation between the values of $\alpha_{1}$ and $\alpha_{2}$. The first are expressed by the norm of the 3-2-1 sequence of Euler angles of the error matrix of each inertial attitude, that is, the product between the true value by the transpose of the estimated value, and are shown in Figure 5. The second are expressed by the comparison parameters $\phi_{AC}$, $\phi_{AD}$, $\phi_{BC}$, and $\phi_{BD}$ as defined in (18). The third are expressed as the cosine of their difference, i.e., $\cos\left(\alpha_{1}-\alpha_{2}\right)$. Both are shown together in Figure 6.

In the ambiguous configuration, at about 25 s, $\phi_{AC}\approx 0$ and $\phi_{BD}\approx 0$. Therefore, near that instant both ${\left(R_{1}^{I}\right)}_{A}\approx {\left(R_{1}^{I}\right)}_{C}$ and ${\left(R_{1}^{I}\right)}_{B}\approx {\left(R_{1}^{I}\right)}_{D}$, which means that the estimated attitude may jump between both solutions, due to the noise, which does happen and is visible in Figure 5. Notice that there is no significant change in the error magnitude before or after the ambiguous configuration, because it is a matter of information symmetry, whereas the degenerate configurations result from a loss of information about a component of the coordinate frame. Moreover, in this case the value of $\alpha_{1}-\alpha_{2}$ satisfies (45), i.e., $\cos\left(\alpha_{1}-\alpha_{2}\right)\approx \pm 1$, which can be seen in Figure 6.

In the degenerate configuration, at about 75 s, the error magnitude of the estimate for $R_{2}^{1}$ increases gradually as the degeneracy gets closer, until it raises sharply approximately at the 75 s mark. This specific degenerate configuration is given by $\;{\;}^{I}\;d_{2/1}=-\;{\;}^{I}\;d_{2}$, which from (22) implies that there is a unique solution for $R_{1}^{I}$, despite there being infinite solutions for $R_{2}^{1}$. This observation, justifies both the values of $\phi_{AC}$ and $\phi_{BC}$ as seen in Figure 6 and the fact that while the error in $R_{2}^{1}$ increases the other errors remain at the same magnitude as observed in Figure 5. Moreover, since (11a) may not be valid, then a rotation compatible with the constraints is chosen in such a case, for example, selecting a value that satisfies both (9a) and (9b).

## 6. Conclusions

In this paper, we analyzed the uniqueness of the solution for the attitude determination problem in a three-vehicle heterogeneous formation, considering both the branches and the entire formation. With respect to the entire formation, there are three possible configurations: degenerate, ambiguous, or regular configurations, respectively, with infinite, two, or one solution. With respect to a single branch, there are also three possibilities: degenerate, ambiguous, or unambiguous branches, respectively, with infinite, two, or one solution. However, the general case is the ambiguous branch. Furthermore, we found the conditions which characterize the ambiguous and degenerate configurations, and branches, thus enabling their detection without solving the attitude problem. We also showed that the measure of the subset of ambiguous configurations is a zero-measure set. Finally, we simulated a maneuver that reaches both a degenerate and an ambiguous configuration and validates the conclusions in this paper. The identification of degenerate and ambiguous configurations is a valuable information for the design of estimators, applicable to this formation, because such configurations introduce challenges to the error stability. Therefore, these results represent one more step towards the design of attitude estimators for three vehicle heterogeneous formations.

## Figures and Tables

**Table 1:** Number of solutions for the entire formation.

		Degenerate			Unambiguous	Ambiguous
		$d_{1}=\pm d_{1/2}$	$d_{2}=\pm d_{2/1}$	$\;{\;}^{I}\;d_{1}=\pm \;{\;}^{I}\;d_{2}$	$d_{1}^{T}S\left(d_{1/2}\right)R_{2}^{1}d_{2}=0$	Otherwise
**Degenerate**	$d_{1}=\pm d_{1/3}$	∞	∞	∞	1	2
	$d_{3}=\pm d_{3/1}$	∞	∞	∞	∞	∞
	$\;{\;}^{I}\;d_{1}=\pm \;{\;}^{I}\;d_{3}$	∞	∞	∞	1	2
**Unambiguous**	$d_{1}^{T}S\left(d_{1/3}\right)R_{3}^{1}d_{3}=0$	1	∞	1	1	1
**Ambiguous**	**Otherwise**	2	∞	2	1	1or2

## Abbreviations

The following abbreviations are used in this manuscript:

TRIAD	Tri-Axial Attitude Determination
QUEST	Quaternion Estimator
FLAE	Fast linear quaternion attitude estimator
LOS	Line-of-sight
GPS	Global Positioning System
SVD	Singular value decomposition

## Appendix A. Bisecting Plane Properties

**Lemma** **A1.**
*Consider a,b,c,d∈S(2), k∈Z, and λ∈R such that*

(A1) λ≠2πk,

(A2) $$b=R\left(\lambda ,a\right)c,$$

(A3) $$d^{T}b=d^{T}c,$$

*and*

(A4) $$a^{T}b=a^{T}c=a^{T}d=0.$$

*Then,*

(A5) $$d=R\left(\frac{\lambda }{2}+k\pi ,a\right)c$$

*with k∈Z.*

**Proof.** Rewrite (A3) by substituting (A2), which gives

(A6) $$d^{T}c=d^{T}R\left(\lambda ,a\right)c.$$

Since (A4) implies that b, c, and d are all coplanar and orthogonal to a, then, for some α∈R, it follows that

(A7) $$d=R\left(\alpha ,a\right)c.$$

Next, substituting (A7) in (A6) gives

$$c^{T}R\left(-\alpha ,a\right)c=c^{T}R\left(\lambda -\alpha ,a\right)c,$$

which applying (2) results in

(A8) $$\begin{array}{c} c^{T}\left[\cos\left(-\alpha \right)I+\left(1-\cos\left(-\alpha \right)\right)a{\left(a\right)}^{T}-\sin\left(-\alpha \right)\;S\left(a\right)\right]c=\\ \;c^{T}\left[\cos\left(\lambda -\alpha \right)I+\left(1-\cos\left(\lambda -\alpha \right)\right)a{\left(a\right)}^{T}-\sin\left(\lambda -\alpha \right)\;S\left(a\right)\right]c. \end{array}$$

Recall that c∈S(2) implies that $c^{T}c=1$. Further recall that $\cos\left(-\alpha \right)=\cos\left(\alpha \right)$ and that (A4) implies both $a^{T}c=0$ and $c^{T}S\left(a\right)c=0$. Then, (A8) is given as

(A9) $$\cos\left(\alpha \right)=\cos\left(\lambda -\alpha \right).$$

Applying the inverse cosine function to both sides of (A9) yields

$$\alpha =\pm \left(\lambda -\alpha \right)+2\pi k\;,k\in Z.$$

Analyzing each sign separately, consider first that α=λ−α+2kπ, which after some rearrangements results in

(A10) $$\alpha =\frac{\lambda }{2}+\pi k.$$

Finally, analyzing the other sign, consider that α=−λ+α+2kπ. It follows that

λ=2πk,

which is excluded from the solutions of (A9) by (A1). Thus, the proof is complete by substituting (A10) in (A7) which yields (A5). □

**Lemma** **A2.**
*Let a, b, c, d∈S(2). If*

(A11) $$a^{T}b=a^{T}c$$

*and*

(A12) $$d^{T}b=d^{T}c$$

*then,*

(A13) $$d^{T}\left[R\left(\psi ,\frac{S\left(a\right)b}{∥S\left(a\right)b∥}\right)b\right]=d^{T}\left[R\left(\psi ,\frac{S\left(a\right)c}{∥S\left(a\right)c∥}\right)c\right]\;,\;\forall \;\psi \in R$$

**Proof.** Take the left side of (A13) and substitute (2), which gives

$$\begin{array}{c} d^{T}\left[R\left(\psi ,\frac{S\left(a\right)b}{∥S\left(a\right)b∥}\right)b\right]\\ \;=d^{T}\left[\cos\psi I+\left(1-\cos\psi \right)\frac{S\left(a\right)b}{∥S\left(a\right)b∥}{\left(\frac{S\left(a\right)b}{∥S\left(a\right)b∥}\right)}^{T}-\sin\psi \;S\left(\frac{S\left(a\right)b}{∥S\left(a\right)b∥}\right)\right]b\;. \end{array}$$

Since $b^{T}S\left(a\right)b=0$ and, from the vector triple product, $S\left(S\left(a\right)b\right)b=-b^{T}ba+b^{T}ab$, it follows that

(A14) $$d^{T}\left[R\left(\psi ,\frac{S\left(a\right)b}{∥S\left(a\right)b∥}\right)b\right]=\left[\cos\left(\psi \right)d^{T}b-\sin\left(\psi \right)d^{T}\frac{-b^{T}ba+b^{T}ab}{∥S\left(a\right)b∥}\right].$$

Substituting (A11) and (A12) and $b^{T}b=1=c^{T}c$ in (A14) gives

(A15) $$d^{T}\left[R\left(\psi ,\frac{S\left(a\right)b}{∥S\left(a\right)b∥}\right)b\right]=\left[\cos\left(\psi \right)d^{T}c-\sin\left(\psi \right)d^{T}\frac{-c^{T}ca+c^{T}ac}{∥S\left(a\right)b∥}\right].$$

Next, using the norm definition, write $∥S\left(a\right)b∥$ as $∥S\left(a\right)b∥=\sqrt{{\left(S\left(a\right)b\right)}^{T}S\left(a\right)b}$ and apply the vector triple product yields

$$∥S\left(a\right)b∥=\sqrt{-b^{T}\left(a^{T}ba-a^{T}ab\right)}=\sqrt{-a^{T}bb^{T}a+a^{T}ab^{T}b}.$$

From (A11) and $b^{T}b=1=c^{T}c$, it follows that

$$∥S\left(a\right)b∥=\sqrt{-a^{T}cc^{T}a+a^{T}ac^{T}c}=\sqrt{-c^{T}\left(a^{T}ca-a^{T}ac\right)},$$

which applying the vector triple product, followed by the norm definition, gives

(A16) $$∥S\left(a\right)b∥=∥S\left(a\right)c∥.$$

Finally, substitute (A16) in (A15) and recall that $\left(1-\cos\psi \right)d^{T}\frac{S\left(a\right)c}{∥S\left(a\right)c∥}{\left(\frac{S\left(a\right)c}{∥S\left(a\right)c∥}\right)}^{T}c=0$. It follows that (A15) is expressed as

$$\begin{array}{c} d^{T}\left[R\left(\psi ,\frac{S\left(a\right)b}{∥S\left(a\right)b∥}\right)b\right]=\\ \;d^{T}\left[\cos\psi I+\left(1-\cos\psi \right)\frac{S\left(a\right)c}{∥S\left(a\right)c∥}{\left(\frac{S\left(a\right)c}{∥S\left(a\right)c∥}\right)}^{T}-\sin\psi \;S\left(\frac{S\left(a\right)c}{∥S\left(a\right)c∥}\right)\right]c, \end{array}$$

which from (2) gives (A13), thus completing the proof. □

**Lemma** **A3.**
*Let a, b, c, d∈S(2). If*

(A17) aTc=aTd,

*and*

(A18) $$b^{T}c=b^{T}d,$$

*Then,*

(A19) $${\left[R\left(\beta ,\frac{S\left(a\right)b}{∥S\left(a\right)b∥}\right)b\right]}^{T}c={\left[R\left(\beta ,\frac{S\left(a\right)b}{∥S\left(a\right)b∥}\right)b\right]}^{T}d\;,\;\forall \;\beta \in R$$

**Proof.** This proof follows similarly to the one from Lemma A2. That is, take the left side of (A19), then apply the rotation definition, the triple vector product, and recall conditions (A17) and (A18) to show that it yields the right side of (A19). Therefore, the details of the proof are omitted. □

## Appendix B. Technical Lemma

This appendix contains the proof for Lemma 1, which is reproduced below.

**Lemma** **A4.**
*Consider $c_{s_{12}}$ and $c_{c_{12}}$ as defined in *(12)*. Then, both*

(A20a) $$c_{s_{12}}=0$$

*and*

(A20b) $$c_{c_{12}}=0$$

*if and only if either*

(A21) d1=±d1/2.

*or*

(A22) $$d_{2}=\pm d_{2/1}.$$

**Proof.** First, assume that (A21) is true. Since $S\left(x\right)x=0$, for any $x\in R^{3}$, then substituting (A21) in (12) yields (A20a) and (A20b). Next, assume that (A22) is verified and left multiply it by $R\left(\theta_{1},n_{1}\right)$, with parameters defined in (10), which gives $R\left(\theta_{1},n_{1}\right)d_{2}=\pm R\left(\theta_{1},n_{1}\right)d_{2/1}$. Recalling (13) and that, by definition, $-d_{1/2}=R\left(\theta_{1},n_{1}\right)d_{2/1}$ [13], it follows that (A22) is equivalent to

(A23) $$d_{2}^{🟉}=\mp d_{1/2}.$$

Then, from $S\left(x\right)x=0$ and substituting (A23) into (12) yields (A20a) and (A20b). Next, consider the converse statement, i.e., that both (A20a) and (A20b) are verified. Let $a,b,c\in R^{3}$, then $a^{T}S\left(b\right)c=0$ if and only if a, b, and c are coplanar or any of these vectors is zero. Then, $c_{s12}$ and $c_{c12}$ can be expressed as $a^{T}S\left(b\right)c$ and $a^{T}S\left(b\right)c^{\prime }$, respectively with $a=d_{1}$, $b=-d_{1/2}$, $c=d_{2}^{🟉}$, and $c^{\prime }=S\left(-d_{1/2}\right)d_{2}^{🟉}$. Consider that $c^{\prime }=0$, which means that (A21) is verified, then both $c_{s12}=0$ and $c_{c12}=0$ because $S\left(x\right)x=0$. Otherwise, consider that $c^{\prime }\neq 0$, which means that c and $c^{\prime }$ are orthogonal. Since verifying both $c_{s12}=0$ and $c_{c12}=0$ implies that a, b, and c are coplanar, while a, b, and $c^{\prime }$ are coplanar, then a and b must be collinear because c and $c^{\prime }$ are orthogonal. Therefore, in this case, (A23) is verified which was previously shown to be equivalent to (A22) and thus concludes the proof. □

## Appendix C. Relation between Measurements of the Same Branch

This appendix shows both the relation between ${\left(\;{\;}^{I}\;d_{1/2}\right)}^{⊥}$ and ${\left(\;{\;}^{I}\;d_{2}\right)}^{⊥}$, and the relation between ${\left(\;{\;}^{I}\;d_{1/3}\right)}^{⊥}$ and ${\left(\;{\;}^{I}\;d_{3}\right)}^{⊥}$. Since the derivation of such relations is analogous in the four ensuing lemmas, only the first lemma is accompanied by its proof.

**Lemma** **A5.**
*Recall the definition of ${\left(R_{1}^{I}\right)}_{A}$, $\theta_{A}$, and $\theta_{B}$ in *(30)*. Further recall the vector transformation in *(36)*. Suppose that both branches of the formation are ambiguous, as defined in Theorem 1, and assume that $R_{1}^{I}={\left(R_{1}^{I}\right)}_{A}$. Then*

(A24) $${\left(\;{\;}^{I}\;d_{1/2}\right)}^{⊥}=R\left(\frac{\theta_{A}-\theta_{B}}{2}+\pi k,\;{\;}^{I}\;d_{1}\right){\left(\;{\;}^{I}\;d_{2}\right)}^{⊥}.$$

**Proof.** First, substitute (33) in $\;{\;}^{I}\;d_{1}^{T}\;\;{\;}^{I}\;d_{2}$, which implies that

Id1TRθA,Id1d2A∗=Id1TRθB,Id1d2B∗.

or, equivalently, from (4),

(A25) $$\;{\;}^{I}\;d_{1}^{T}d_{2A}^{*}=\;{\;}^{I}\;d_{1}^{T}d_{2B}^{*}.$$

Similarly, consider the inner product given by ${\left(R_{1}^{I}d_{1/2}\right)}^{T}\;{\;}^{I}\;d_{2}$, which from (30) and (33) implies that

$${\left(R\left(\theta_{A},\;{\;}^{I}\;d_{1}\right)R_{}^{*}d_{1/2}\right)}^{T}R\left(\theta_{A},\;{\;}^{I}\;d_{1}\right)d_{2A}^{*}={\left(R\left(\theta_{B},\;{\;}^{I}\;d_{1}\right)R_{}^{*}d_{1/2}\right)}^{T}R\left(\theta_{B},\;{\;}^{I}\;d_{1}\right)d_{2B}^{*},$$

or, equivalently,

(A26) $${\left(R_{}^{*}d_{1/2}\right)}^{T}d_{2A}^{*}={\left(R_{}^{*}d_{1/2}\right)}^{T}d_{2B}^{*}.$$

Next, consider the angle $\psi_{1}\in R$. Then, apply Lemma A2, in Appendix A, with $a=\;{\;}^{I}\;d_{1}$, $b=d_{2A}^{*}$, $c=d_{2B}^{*}$, and $d=R_{}^{*}d_{1/2}$, recalling that in such case (A11) and (A12) are given respectively by (A25) and (A26). It follows that

(A27) $${\left(R_{}^{*}d_{1/2}\right)}^{T}R\left(\psi_{1},m_{d_{2A}^{*}}\right)d_{2A}^{*}={\left(R_{}^{*}d_{1/2}\right)}^{T}R\left(\psi_{1},m_{d_{2B}^{*}}\right)d_{2B}^{*},$$

where $m_{d_{2A}^{*}}$ and $m_{d_{2B}^{*}}$ are defined in (35). Since $\psi_{1}$ is arbitrary, recall (36) and choose $\psi_{1}$ such that (A27) is expressed as

(A28) $${\left(R_{}^{*}d_{1/2}\right)}^{T}{\left(d_{2A}^{*}\right)}^{⊥}={\left(R_{}^{*}d_{1/2}\right)}^{T}{\left(d_{2B}^{*}\right)}^{⊥}.$$

Similarly, consider the angle $\psi_{2}\in R$ and recall from (37) that, by definition,

(A29) $$\;{\;}^{I}\;d_{1}^{T}{\left(d_{2A}^{*}\right)}^{⊥}=\;{\;}^{I}\;d_{1}^{T}{\left(d_{2B}^{*}\right)}^{⊥}=0.$$

Then, apply Lemma A3, in Appendix A, with $a=\;{\;}^{I}\;d_{1}$, $b=R_{}^{*}d_{1/2}$, $c={\left(d_{2A}^{*}\right)}^{⊥}$, and $d={\left(d_{2B}^{*}\right)}^{⊥}$. Recall that in such case (A17) and (A18) are given respectively by (A29) and (A28). It follows that

$${\left(R\left(\psi_{2},m_{R_{}^{*}d_{1/2}}\right)R_{}^{*}d_{1/2}\right)}^{T}{\left(d_{2A}^{*}\right)}^{⊥}={\left(R\left(\psi_{2},m_{R_{}^{*}d_{1/2}}\right)R_{}^{*}d_{1/2}\right)}^{T}{\left(d_{2B}^{*}\right)}^{⊥},$$

where $m_{R_{}^{*}d_{1/2}}$ is defined in (35). Then, recalling (36) and choosing the appropriate value for $\psi_{2}$ gives

(A30) $${\left[{\left(R_{}^{*}d_{1/2}\right)}^{⊥}\right]}^{T}{\left(d_{2A}^{*}\right)}^{⊥}={\left[{\left(R_{}^{*}d_{1/2}\right)}^{⊥}\right]}^{T}{\left(d_{2B}^{*}\right)}^{⊥}.$$

Next, from (37), recall that

(A31) $$\;{\;}^{I}\;d_{1}^{T}{\left(d_{2A}^{*}\right)}^{⊥}=\;{\;}^{I}\;d_{1}^{T}{\left(d_{2B}^{*}\right)}^{⊥}=\;{\;}^{I}\;d_{1}^{T}{\left(R_{}^{*}d_{1/2}\right)}^{⊥}=0$$

and from (33) that

(A32) $$R\left(-\theta_{A},\;{\;}^{I}\;d_{1}\right)\;{\;}^{I}\;d_{2}=d_{2A}^{*}=R\left(\theta_{B}-\theta_{A},\;{\;}^{I}\;d_{1}\right)d_{2B}^{*}.$$

Moreover, expanding ${\left(d_{2A}^{*}\right)}^{⊥}$ with (36) gives

$${\left(d_{2A}^{*}\right)}^{⊥}=R\left(\psi ,m_{d_{2A}^{*}}\right)d_{2A}^{*},$$

which is rewritten, by applying (A32), as

(A33) $${\left(d_{2A}^{*}\right)}^{⊥}=R\left(\psi ,m_{d_{2A}^{*}}\right)R\left(\theta_{B}-\theta_{A},\;{\;}^{I}\;d_{1}\right)d_{2B}^{*}.$$

Afterwards, recall the definition (35) and consider the numerator of $m_{d_{2A}^{*}}$. It follows from (7) and (A32) that

(A34) $$S\left(\;{\;}^{I}\;d_{1}\right)d_{2A}^{*}=S\left(\;{\;}^{I}\;d_{1}\right)R\left(\theta_{B}-\theta_{A},\;{\;}^{I}\;d_{1}\right)d_{2B}^{*}=R\left(\theta_{B}-\theta_{A},\;{\;}^{I}\;d_{1}\right)S\left(\;{\;}^{I}\;d_{1}\right)d_{2B}^{*}.$$

Meanwhile, consider the denominator of $m_{d_{2A}^{*}}$. It follows, from (A32), that

$$∥S\left(\;{\;}^{I}\;d_{1}\right)d_{2A}^{*}∥=∥S\left(\;{\;}^{I}\;d_{1}\right)R\left(\theta_{B}-\theta_{A},\;{\;}^{I}\;d_{1}\right)d_{2B}^{*}∥\;,$$

and then, from (3) and (7), that

(A35) $$∥S\left(\;{\;}^{I}\;d_{1}\right)d_{2A}^{*}∥=∥R\left(\theta_{B}-\theta_{A},\;{\;}^{I}\;d_{1}\right)S\left(\;{\;}^{I}\;d_{1}\right)d_{2B}^{*}∥=∥S\left(\;{\;}^{I}\;d_{1}\right)d_{2B}^{*}∥.$$

Consequently, recalling the definition of both $m_{d_{2A}^{*}}$ and $m_{d_{2B}^{*}}$ in (35), it is concluded that (A34) and (A35) imply

(A36) $$m_{d_{2A}^{*}}=R\left(\theta_{B}-\theta_{A},\;{\;}^{I}\;d_{1}\right)m_{d_{2B}^{*}}.$$

Therefore, recalling (6), it follows that (A33) is also given as

$${\left(d_{2A}^{*}\right)}^{⊥}=R\left(\theta_{B}-\theta_{A},\;{\;}^{I}\;d_{1}\right)R\left(\psi ,R\left(\theta_{B}-\theta_{A},\;{\;}^{I}\;d_{1}\right)m_{d_{2A}^{*}}\right)d_{2B}^{*},$$

which, from (A36), yields

$${\left(d_{2A}^{*}\right)}^{⊥}=R\left(\theta_{B}-\theta_{A},\;{\;}^{I}\;d_{1}\right)R\left(\psi ,m_{d_{2B}^{*}}\right)d_{2B}^{*},$$

or, equivalently, from (36),

(A37) $${\left(d_{2A}^{*}\right)}^{⊥}=R\left(\theta_{B}-\theta_{A},\;{\;}^{I}\;d_{1}\right){\left(d_{2B}^{*}\right)}^{⊥}.$$

Next, consider that $\theta_{A}-\theta_{B}\neq 2\pi k$, with k∈Z. Then, the conditions for Lemma A1, in Appendix A given by (A2), (A3) and (A4), are respectively verified by (A37), (A30) and (A31), with $\lambda =\theta_{B}-\theta_{A}$, $a=\;{\;}^{I}\;d_{1}$, $b={\left(d_{2A}^{*}\right)}^{⊥}$, $c={\left(d_{2B}^{*}\right)}^{⊥}$, and $d={\left(R_{}^{*}d_{1/2}\right)}^{⊥}$. Therefore, it follows that

(A38) $${\left(R_{}^{*}d_{1/2}\right)}^{⊥}=R\left(\frac{\theta_{B}-\theta_{A}}{2}+\pi k,\;{\;}^{I}\;d_{1}\right){\left(d_{2B}^{*}\right)}^{⊥}.$$

Next, assume that $R_{1}^{I}={\left(R_{1}^{I}\right)}_{A}$ and, as a consequence of having two ambiguous branches, that $R_{2}^{1}={\left(R_{2}^{1}\right)}_{A}$. Then, apply (7) and (31a) to $R\left(\theta_{A},\;{\;}^{I}\;d_{1}\right)S\left(\;{\;}^{I}\;d_{1}\right)d_{2A}^{*}$, which gives

$$R\left(\theta_{A},\;{\;}^{I}\;d_{1}\right)S\left(\;{\;}^{I}\;d_{1}\right)d_{2A}^{*}=S\left(\;{\;}^{I}\;d_{1}\right)R\left(\theta_{A},\;{\;}^{I}\;d_{1}\right)R_{}^{*}{\left(R_{2}^{1}\right)}_{A}d_{2}$$

or, equivalently, from (14b) and (30a), $R_{1}^{I}={\left(R_{1}^{I}\right)}_{A}$, and $R_{2}^{1}={\left(R_{2}^{1}\right)}_{A}$,

(A39) $$R\left(\theta_{A},\;{\;}^{I}\;d_{1}\right)S\left(\;{\;}^{I}\;d_{1}\right)d_{2A}^{*}=S\left(\;{\;}^{I}\;d_{1}\right)\;{\;}^{I}\;d_{2}.$$

Meanwhile, apply (3) and (7) to $∥S\left(\;{\;}^{I}\;d_{1}\right)d_{2A}^{*}∥$, which gives

$$∥S\left(\;{\;}^{I}\;d_{1}\right)d_{2A}^{*}∥=∥R\left(\theta_{A},\;{\;}^{I}\;d_{1}\right)S\left(\;{\;}^{I}\;d_{1}\right)d_{2A}^{*}∥=∥S\left(\;{\;}^{I}\;d_{1}\right)R\left(\theta_{A},\;{\;}^{I}\;d_{1}\right)d_{2A}^{*}∥.$$

Recalling (31a) and (30a), $R_{1}^{I}={\left(R_{1}^{I}\right)}_{A}$, and $R_{2}^{1}={\left(R_{2}^{1}\right)}_{A}$, it follows that

$$∥S\left(\;{\;}^{I}\;d_{1}\right)d_{2A}^{*}∥=∥S\left(\;{\;}^{I}\;d_{1}\right)R_{1}^{I}R_{2}^{1}d_{2}∥.$$

Finally, substituting (14b) results in

(A40) $$∥S\left(\;{\;}^{I}\;d_{1}\right)d_{2A}^{*}∥=∥S\left(\;{\;}^{I}\;d_{1}\right)\;{\;}^{I}\;d_{2}∥.$$

Therefore, (A39) and (A40) imply that

(A41) $$R\left(\theta_{A},\;{\;}^{I}\;d_{1}\right)m_{d_{2A}^{*}}=m_{\;{\;}^{I}\;d_{2}},$$

where $m_{d_{2A}^{*}}$ and $m_{\;\;{\;}^{I}\;d_{2}}$ are defined in (35). Following a similar train of thought, while recalling that by definition $\;{\;}^{I}\;d_{1/2}=R_{1}^{I}d_{1/2}$, it follows that

(A42) $$R\left(\theta_{A},\;{\;}^{I}\;d_{1}\right)m_{R_{}^{*}d_{1/2}}=m_{\;\;{\;}^{I}\;d_{1/2}},$$

with $m_{R_{}^{*}d_{1/2}}$ and $m_{\;\;{\;}^{I}\;d_{1/2}}$ given as defined in (35). Next, left multiplying (A38) by $R\left(\theta_{A},\;{\;}^{I}\;d_{1}\right)$, while recalling (36) with appropriate $\psi_{3},\psi_{4}\in R$, gives

$$\begin{array}{c} R\left(\theta_{A},\;{\;}^{I}\;d_{1}\right)R\left(\psi_{3},m_{R_{}^{*}d_{1/2}}\right)R_{}^{*}d_{1/2}\\ \;=R\left(\theta_{A},\;{\;}^{I}\;d_{1}\right)R\left(\frac{\theta_{A}-\theta_{B}}{2}+\pi k,\;{\;}^{I}\;d_{1}\right)R\left(\psi_{4},m_{d_{2A}^{*}}\right)d_{2A}^{*}, \end{array}$$

which from (4) and (6) becomes

$$\begin{array}{c} R\left(\theta_{A},\;{\;}^{I}\;d_{1}\right)R\left(\psi_{3},m_{R_{}^{*}d_{1/2}}\right)R_{}^{*}d_{1/2}\\ \;=R\left(\frac{\theta_{A}-\theta_{B}}{2}+\pi k,\;{\;}^{I}\;d_{1}\right)R\left(\theta_{A},\;{\;}^{I}\;d_{1}\right)R\left(\psi_{4},m_{d_{2A}^{*}}\right)d_{2A}^{*}. \end{array}$$

Applying (6), it follows that

$$\begin{array}{c} R\left(\psi_{3},R\left(\theta_{A},\;{\;}^{I}\;d_{1}\right)m_{R_{}^{*}d_{1/2}}\right)R\left(\theta_{A},\;{\;}^{I}\;d_{1}\right)R_{}^{*}d_{1/2}\\ \;=R\left(\frac{\theta_{A}-\theta_{B}}{2}+\pi k,\;{\;}^{I}\;d_{1}\right)R\left(\psi_{4},R\left(\theta_{A},\;{\;}^{I}\;d_{1}\right)m_{d_{2A}^{*}}\right)R\left(\theta_{A},\;{\;}^{I}\;d_{1}\right)d_{2A}^{*}, \end{array}$$

which recalling (A41) and (A42), becomes

$$\begin{array}{c} R\left(\psi_{3},m_{\;\;{\;}^{I}\;d_{1/2}}\right)R\left(\theta_{A},\;{\;}^{I}\;d_{1}\right)R_{}^{*}d_{1/2}\\ \;=R\left(\frac{\theta_{A}-\theta_{B}}{2}+\pi k,\;{\;}^{I}\;d_{1}\right)R\left(\psi_{4},m_{\;\;{\;}^{I}\;d_{2}}\right)R\left(\theta_{A},\;{\;}^{I}\;d_{1}\right)d_{2A}^{*}, \end{array}$$

or, equivalently, from (30a) and (31a),

(A43) $$\begin{array}{c} R\left(\psi_{3},m_{\;\;{\;}^{I}\;d_{1/2}}\right){\left(R_{1}^{I}\right)}_{A}d_{1/2}\\ \;=R\left(\frac{\theta_{A}-\theta_{B}}{2}+\pi k,\;{\;}^{I}\;d_{1}\right)R\left(\psi_{4},m_{\;\;{\;}^{I}\;d_{2}}\right){\left(R_{1}^{I}\right)}_{A}{\left(R_{2}^{1}\right)}_{A}d_{2}. \end{array}$$

Furthermore, recall the assumption that $R_{1}^{I}={\left(R_{1}^{I}\right)}_{A}$ and $R_{2}^{1}={\left(R_{2}^{1}\right)}_{A}$, then (14b) and $\;{\;}^{I}\;d_{1/2}=R_{1}^{I}d_{1/2}$ imply that (A43) can be rewritten as

(A44) $$R\left(\psi_{3},m_{\;\;{\;}^{I}\;d_{1/2}}\right)\;{\;}^{I}\;d_{1/2}=R\left(\frac{\theta_{A}-\theta_{B}}{2}+\pi k,\;{\;}^{I}\;d_{1}\right)R\left(\psi_{4},m_{\;\;{\;}^{I}\;d_{2}}\right)\;{\;}^{I}\;d_{2}.$$

Finally, recall that both sides of (A38) satisfy (37) and that (A44) results from left multiplying (A38) by $R\left(\theta_{A},\;{\;}^{I}\;d_{1}\right)$. Therefore, both sides of (A44) also satisfy (37), which means that applying (36) to (A44) yields (A24) and concludes the proof. □

**Lemma** **A6.**
*Recall the definition of ${\left(R_{1}^{I}\right)}_{B}$, $\theta_{A}$, and $\theta_{B}$ in *(30)*. Further recall the vector transformation in *(36)*. Suppose that both branches of the formation are ambiguous, as defined in Theorem 1 and assume that $R_{1}^{I}={\left(R_{1}^{I}\right)}_{B}$. Then*

$${\left(\;{\;}^{I}\;d_{1/2}\right)}^{⊥}=R\left(-\frac{\theta_{A}-\theta_{B}}{2}+\pi k,\;{\;}^{I}\;d_{1}\right){\left(\;{\;}^{I}\;d_{2}\right)}^{⊥}.$$

**Lemma** **A7.**
*Recall the definition of ${\left(R_{1}^{I}\right)}_{C}$, $\theta_{C}$, and $\theta_{D}$ in *(30)*. Further recall the vector transformation in *(36)*. Suppose that both branches of the formation are ambiguous, as defined in Theorem 1 and assume that $R_{1}^{I}={\left(R_{1}^{I}\right)}_{C}$. Then*

$${\left(\;{\;}^{I}\;d_{1/3}\right)}^{⊥}=R\left(\frac{\theta_{C}-\theta_{D}}{2}+\pi k,\;{\;}^{I}\;d_{1}\right){\left(\;{\;}^{I}\;d_{3}\right)}^{⊥}.$$

**Lemma** **A8.**
*Recall the definition of ${\left(R_{1}^{I}\right)}_{D}$, $\theta_{C}$, and $\theta_{D}$ in *(30)*. Further recall the vector transformation in *(36)*. Suppose that both branches of the formation are ambiguous, as defined in Theorem 1 and assume that $R_{1}^{I}={\left(R_{1}^{I}\right)}_{D}$. Then*

$${\left(\;{\;}^{I}\;d_{1/3}\right)}^{⊥}=R\left(-\frac{\theta_{C}-\theta_{D}}{2}+\pi k,\;{\;}^{I}\;d_{1}\right){\left(\;{\;}^{I}\;d_{3}\right)}^{⊥}.$$

## Appendix D. Relation between Same Type of Measurement

This appendix shows both the relation between ${\left(\;{\;}^{I}\;d_{2}\right)}^{⊥}$ and ${\left(\;{\;}^{I}\;d_{3}\right)}^{⊥}$, and the relation between ${\left(\;{\;}^{I}\;d_{1/2}\right)}^{⊥}$ and ${\left(\;{\;}^{I}\;d_{1/3}\right)}^{⊥}$. Since the derivation of such relations is analogous in both ensuing lemmas, only the first lemma is accompanied by its proof.

**Lemma** **A9.**
*Recall the relation between $\;{\;}^{I}\;d_{3}$ and $\;{\;}^{I}\;d_{2}$ defined in *(29a)* and the vector transformation in *(36)*. Then,*

(A45) $${\left(\;{\;}^{I}\;d_{3}\right)}^{⊥}=R\left(\alpha_{1}+\pi k_{1},\;{\;}^{I}\;d_{1}\right){\left(\;{\;}^{I}\;d_{2}\right)}^{⊥},$$

*with $\alpha_{1}$ defined in *(29a)* and $k_{1}\in Z$.*

**Proof.** First, consider the definition of $\;{\left(\;{\;}^{I}\;d_{3}\right)}^{⊥}$ in (36), with an appropriate ψ∈R, and substitute (29a), which gives

(A46) $${\left(\;{\;}^{I}\;d_{3}\right)}^{⊥}=R\left(\psi ,\frac{S\left(\;{\;}^{I}\;d_{1}\right)R\left(\alpha_{1},\;{\;}^{I}\;d_{1}\right)R\left(\beta ,m_{\;{\;}^{I}\;d_{2}}\right)\;{\;}^{I}\;d_{2}}{∥S\left(\;{\;}^{I}\;d_{1}\right)R\left(\alpha_{1},\;{\;}^{I}\;d_{1}\right)R\left(\beta ,m_{\;{\;}^{I}\;d_{2}}\right)\;{\;}^{I}\;d_{2}∥}\right)R\left(\alpha_{1},\;{\;}^{I}\;d_{1}\right)R\left(\beta ,m_{\;{\;}^{I}\;d_{2}}\right)\;{\;}^{I}\;d_{2},$$

where $m_{\;{\;}^{I}\;d_{2}}$, as defined in (35), is used to simplify the notation. Then, recall (3) which results from the preservation of the norm of a vector transformed by a rotation. Thus,

$$∥S\left(\;{\;}^{I}\;d_{1}\right)R\left(\alpha_{1},\;{\;}^{I}\;d_{1}\right)R\left(\beta ,m_{\;{\;}^{I}\;d_{2}}\right)\;{\;}^{I}\;d_{2}∥=∥R{\left(\alpha_{1},\;{\;}^{I}\;d_{1}\right)}^{T}S\left(\;{\;}^{I}\;d_{1}\right)R\left(\alpha_{1},\;{\;}^{I}\;d_{1}\right)R\left(\beta ,m_{\;{\;}^{I}\;d_{2}}\right)\;{\;}^{I}\;d_{2}∥\;$$

which means that (A46) is equivalent to

(A47) $$\begin{array}{c} {\left(\;{\;}^{I}\;d_{3}\right)}^{⊥}=R\left(\psi ,\frac{S\left(\;{\;}^{I}\;d_{1}\right)R\left(\alpha_{1},\;{\;}^{I}\;d_{1}\right)R\left(\beta ,m_{\;{\;}^{I}\;d_{2}}\right)\;{\;}^{I}\;d_{2}}{∥R{\left(\alpha_{1},\;{\;}^{I}\;d_{1}\right)}^{T}S\left(\;{\;}^{I}\;d_{1}\right)R\left(\alpha_{1},\;{\;}^{I}\;d_{1}\right)R\left(\beta ,m_{\;{\;}^{I}\;d_{2}}\right)\;{\;}^{I}\;d_{2}∥}\right)\;\\ R\left(\alpha_{1},\;{\;}^{I}\;d_{1}\right)R\left(\beta ,m_{\;{\;}^{I}\;d_{2}}\right)\;{\;}^{I}\;d_{2}. \end{array}$$

Next, apply (6) to (A47), which gives

$$\begin{array}{c} {\left(\;{\;}^{I}\;d_{3}\right)}^{⊥}=R\left(\alpha_{1},\;{\;}^{I}\;d_{1}\right)\\ \;R\left(\psi ,\frac{R{\left(\alpha_{1},\;{\;}^{I}\;d_{1}\right)}^{T}S\left(\;{\;}^{I}\;d_{1}\right)R\left(\alpha_{1},\;{\;}^{I}\;d_{1}\right)R\left(\beta ,m_{\;{\;}^{I}\;d_{2}}\right)\;{\;}^{I}\;d_{2}}{∥R{\left(\alpha_{1},\;{\;}^{I}\;d_{1}\right)}^{T}S\left(\;{\;}^{I}\;d_{1}\right)R\left(\alpha_{1},\;{\;}^{I}\;d_{1}\right)R\left(\beta ,m_{\;{\;}^{I}\;d_{2}}\right)\;{\;}^{I}\;d_{2}∥}\right)R\left(\beta ,m_{\;{\;}^{I}\;d_{2}}\right)\;{\;}^{I}\;d_{2}. \end{array}$$

or, equivalently, from (7),

(A48) $${\left(\;{\;}^{I}\;d_{3}\right)}^{⊥}=R\left(\alpha_{1},\;{\;}^{I}\;d_{1}\right)R\left(\psi ,\frac{S\left(\;{\;}^{I}\;d_{1}\right)R\left(\beta ,m_{\;{\;}^{I}\;d_{2}}\right)\;{\;}^{I}\;d_{2}}{∥S\left(\;{\;}^{I}\;d_{1}\right)R\left(\beta ,m_{\;{\;}^{I}\;d_{2}}\right)\;{\;}^{I}\;d_{2}∥}\right)R\left(\beta ,m_{\;{\;}^{I}\;d_{2}}\right)\;{\;}^{I}\;d_{2}.$$

Since (37) implies that $\;{\;}^{I}\;d_{1}^{T}{\left(\;{\;}^{I}\;d_{3}\right)}^{⊥}=0$, then

$$\;{\;}^{I}\;d_{1}^{T}R\left(\alpha_{1},\;{\;}^{I}\;d_{1}\right)R\left(\psi ,\frac{S\left(\;{\;}^{I}\;d_{1}\right)R\left(\beta ,m_{\;{\;}^{I}\;d_{2}}\right)\;{\;}^{I}\;d_{2}}{∥S\left(\;{\;}^{I}\;d_{1}\right)R\left(\beta ,m_{\;{\;}^{I}\;d_{2}}\right)\;{\;}^{I}\;d_{2}∥}\right)R\left(\beta ,m_{\;{\;}^{I}\;d_{2}}\right)\;{\;}^{I}\;d_{2}=0$$

or, equivalently, from (4),

(A49) $$\;{\;}^{I}\;d_{1}^{T}R\left(\psi ,\frac{S\left(\;{\;}^{I}\;d_{1}\right)R\left(\beta ,m_{\;{\;}^{I}\;d_{2}}\right)\;{\;}^{I}\;d_{2}}{∥S\left(\;{\;}^{I}\;d_{1}\right)R\left(\beta ,m_{\;{\;}^{I}\;d_{2}}\right)\;{\;}^{I}\;d_{2}∥}\right)R\left(\beta ,m_{\;{\;}^{I}\;d_{2}}\right)\;{\;}^{I}\;d_{2}=0.$$

Therefore, $R\left(\psi ,\frac{S\left(\;{\;}^{I}\;d_{1}\right)R\left(\beta ,m_{\;{\;}^{I}\;d_{2}}\right)\;{\;}^{I}\;d_{2}}{∥S\left(\;{\;}^{I}\;d_{1}\right)R\left(\beta ,m_{\;{\;}^{I}\;d_{2}}\right)∥\;{\;}^{I}\;d_{2}}\right)R\left(\beta ,m_{\;{\;}^{I}\;d_{2}}\right)\;{\;}^{I}\;d_{2}$ can be written as ${\left(\;{\;}^{I}\;d_{2}\right)}^{⊥}$, because the axis of the rotation on the left is orthogonal to the plane given by $\;{\;}^{I}\;d_{1}$ and $\;{\;}^{I}\;d_{2}$ and therefore $\frac{S\left(\;{\;}^{I}\;d_{1}\right)R\left(\beta ,m_{\;{\;}^{I}\;d_{2}}\right)\;{\;}^{I}\;d_{2}}{∥S\left(\;{\;}^{I}\;d_{1}\right)R\left(\beta ,m_{\;{\;}^{I}\;d_{2}}\right)∥\;{\;}^{I}\;d_{2}}=\pm m_{\;{\;}^{I}\;d_{2}}$. Hence, (A48) is rewritten as

$${\left(\;{\;}^{I}\;d_{3}\right)}^{⊥}=R\left(\alpha_{1}+\pi k_{1},\;{\;}^{I}\;d_{1}\right){\left(\;{\;}^{I}\;d_{2}\right)}^{⊥},$$

with $k_{1}\in Z$, because there are two possible vectors in S(2) which are orthogonal to $\;{\;}^{I}\;d_{1}$. Thus, the proof is complete. □

**Lemma** **A10.**
*Recall the relation between $\;{\;}^{I}\;d_{3}$ and $\;{\;}^{I}\;d_{2}$ defined in *(29a)* and the vector transformation in *(36)*. Then,*

$${\left(\;{\;}^{I}\;d_{1/3}\right)}^{⊥}=R\left(\alpha_{2}+\pi k_{2},\;{\;}^{I}\;d_{1}\right){\left(\;{\;}^{I}\;d_{1/2}\right)}^{⊥}.$$

*with $\alpha_{2}$ defined in *(29b)* and $k_{2}\in Z$.*
